# Finite population distribution function estimation with dual use of auxiliary information under simple and stratified random sampling

**DOI:** 10.1371/journal.pone.0239098

**Published:** 2020-09-28

**Authors:** Sardar Hussain, Sohaib Ahmad, Mariyam Saleem, Sohail Akhtar

**Affiliations:** 1 Department of Statistics, Quaid-I-Azam University, Islamabad, Pakistan; 2 Department of Statistics, Government College Univerisity, Lahore, Pakistan; Tongii University, CHINA

## Abstract

The main purpose of this paper is to propose two new estimators for estimating the finite population distribution function under simple and stratified random sampling schemes using supplementary information on the distribution function, mean and ranks of the auxiliary variable. The mathematical expressions for the bias and mean squared error of the proposed estimators are derived under the first order of approximation. The theoretical and empirical studies showed that the proposed estimators uniformly perform better than the existing estimators in terms of the percentage relative efficiency.

## 1 Introduction

In survey sampling, the use of suitable auxiliary information improves the precision of estimators of the unknown population parameter(s). Several estimators of population parameters, including the population mean, median, total, distribution function, quantiles, etc., exist in the literature, and requires, supplementary information on one or more auxiliary variables along with the information on the study variable. A number of studies have been published on the estimation of the population mean. Some important references to the population mean estimation using auxiliary information include Murthy [1], Sisodia and Dwivedi [2], Srivastava and Jhajj [3], Rao [4], Upadhyaya and Singh [5], Singh [6], Kadilar and Cingi [7], Kadilar and Cingi [8], Gupta and Shabbir [9], Grover and Kaur [10], Grover and Kaur [11], Lu [12], Muneer et al. [13], Shabbir and Gupta [14], and Gupta and Yadav [15]. In these studies, the authors have proposed improved ratio, product, and regression type estimators for estimating the finite population mean. These authors have used a single auxiliary variable for the estimation procedure. In a recent study, Haq et al. [16] suggested using ranks of the auxiliary varible as an additional auxiliary variable to increase the precision of the estimator of the population mean in simple random sampling. However, to the best of our knowledge, there is no study concerning the use of two auxiliary information variables for the estimation of the finite population distribution function.

The problem of estimating the finite population cumulative distribution function (CDF) arises when the interest lies in finding out the proportion of the values of the study variable that are less or equal to a certain value. There are situations where estimating the CDF is deemed necessary. For example, for a nutritionist, it is interesting to know the proportion of the population that consumes 25% or more of the calorie intake from saturated fat. In the literature, many authors have estimated the CDF using information about one or more auxiliary variables. Chambers and Dunstan [17] suggested an estimator for estimating the CDF that requires information both on the study and auxiliary variables. Similarly, Rao et al. [18] and Rao [19] proposed ratio and difference/regression estimators for estimating the CDF under a general sampling design. Kuk [20] suggested a kernel method for estimating the CDF using the auxiliary information. Ahmed and Abu-Dayyeh [21] estimated the CDF using the information on multiple auxiliary variables. Rueda et al. [22] used a calibration approach to develop an estimator for estimating the CDF. Singh et al. [23] considered the problem of estimating the CDF and quantiles with the use of at the estimation stage of a survey. Moreover, Yaqub and Shabbir [24] considered a generalised class of estimators for estimating the CDF in the presence of non-response. Chen and Chen [25] investigated the injury severities of truck drivers in single-and multi-vehicle accidents on rural highways while Zeng et al. [26] worked on a multivariate random-parameter Tobit model for analysing highway crash rates by injury severity, and Yaqub and Shabbir [24] considered a generalised class of estimators for estimating the CDF in the presence of non-response. Dong et al. [27] investigated the differences of single-vehicle and multi-vehicle accident probability using a mixed logit model, Chen et al. [28] worked on an analysis of hourly crash likelihood using an unbalanced panel data mixed logit model and real-time driving environmental big data, Zeng et al. [29] suggested jointly modelling area-level crash rates by severity, and Zeng et al. [30] used spatial joint analysis for zonal daytime and night-time crash frequencies using a Bayesian bivariate conditional autoregressive model. However, these estimators only used one auxiliary variate.

In this paper, we propose two new families of estimators for estimating the CDF using the information on the distribution function, ranks, and mean of the auxiliary variable under simple random sampling and stratified random sampling. The bias and mean squared errors (MSEs) of the existing and proposed estimators of the CDF are derived under the first order of approximation. The theoretical and numerical comparisons showed that the proposed estimators are more precise than the existing adapted estimators when estimating the CDF of a finite population.

## 2 Notation in simple random sampling

Consider a finite population Ω = {1,2,…,*N*} of *N* distinct units. In order to estimate the finite population distribution function, a sample of size *n* units is drawn from Ω using simple random sampling without replacement. Suppose *Y* and *X* are the study and auxiliary variables, respectively. Let Z denote the ranks of *X*, *I*(*Y*≤*y*) is indicator variable based on *Y* and *I*(*X*≤*x*) is indicator variable based on *X*. Similarly, $F(y)=\sum_{i=1}^{N}I(Y_{i}\leq y)/N$ and $\hat{F}(y)=\sum_{i=1}^{n}I(Y_{i}\leq y)/n$ ($F(x)=\sum_{i=1}^{N}I(X_{i}\leq x)/N$ and $\hat{F}(x)=\sum_{i=1}^{n}I(X_{i}\leq x)/n$) are the population and sample distribution functions of *Y* (*X*), respectively. Let $\bar{X}=\sum_{i=1}^{N}X_{i}/N$ and $\hat{\bar{X}}=\sum_{i=1}^{n}X_{i}/n$ ($\bar{Z}=\sum_{i=1}^{N}Z_{i}/N$ and $\hat{\bar{Z}}=\sum_{i=1}^{n}Z_{i}/n$) are the population and sample means of *X* (*Z*), respectively.

In order to obtain the biases and mean squared errors (MSEs) of the adapted and proposed estimators of F(y), we consider the following relative error terms. Let

$$e_{1}=\frac{\hat{F}(y)-F(y)}{F(y)},e_{2}=\frac{\hat{F}(x)-F(x)}{F(x)},e_{3}=\frac{\hat{\bar{X}}-\bar{X}}{\bar{X}}ande_{4}=\frac{\hat{\bar{Z}}-\bar{Z}}{\bar{Z}},$$

such that *e*_*i*_ = 0 for *i* = 0,1,2,3, where *E*(⋅) is the mathematical expectation of (⋅). Let

$$V_{rstu}=E[e_{1}^{r}\;e_{2}^{s}\;e_{3}^{t}\;e_{4}^{u}],$$

$$E(e_{1}^{2})=\lambda C_{1}^{2}=V_{2000},E(e_{2}^{2})=\lambda C_{2}^{2}=V_{0200},E(e_{3}^{2})=\lambda C_{3}^{2}=V_{0020},E(e_{4}^{2})=\lambda C_{4}^{2}=V_{0002},$$

$$E(e_{1}e_{2})=\lambda R_{12}C_{1}C_{2}=V_{1100},E(e_{1}e_{3})=\lambda R_{13}C_{1}C_{3}=V_{1010},E(e_{1}e_{4})=\lambda R_{14}C_{1}C_{4}=V_{1001},$$

$$E(e_{2}e_{3})=\lambda R_{23}C_{2}C_{3}=V_{0110},E(e_{2}e_{4})=\lambda R_{24}C_{2}C_{4}=V_{0101},$$

where *λ* = (*N*−*n*)/(*nN*), $S_{1}^{2}=\sum_{i=1}^{N}{\left(I(Y_{i}\leq y)-F(y)\right)}^{2}/(N-1)$,

$S_{2}^{2}=\sum_{i=1}^{N}{\left(I(X_{i}\leq x)-F(x)\right)}^{2}/(N-1)$,

$S_{3}^{2}=\sum_{i=1}^{N}(X_{i}-\bar{X}{)}^{2}/(N-1)$,

$S_{4}^{2}=\sum_{i=1}^{N}(Z_{i}-\bar{Z}{)}^{2}/(N-1)$,

*C*_1_ = *S*_1_/*F*(*y*), *C*_2_ = *S*_2_/*F*(*x*),

$C_{3}=S_{3}/\bar{X}$, $C_{4}=S_{4}/\bar{Z}$, *R*_12_ = *S*_12_/(*F*(*y*)*F*(*x*)),

$R_{13}=S_{13}/(F(y)\bar{X})$, $R_{23}=S_{23}/(F(y)\bar{Z})$, $R_{14}=S_{14}/(F(x)\bar{X})$, $R_{24}=S_{24}/(F(x)\bar{Z})$, $S_{12}=\sum_{i=1}^{N}\{\left(I(Y_{i}\leq y)-F(y))(I(X_{i}\leq x)-F(x)\right)\}/(N-1)$,

$S_{13}=\sum_{i=1}^{N}\{\left(I(Y_{i}\leq y)-F(y))(X_{i}-\bar{X}\right)\}/(N-1)$,

$S_{23}=\sum_{i=1}^{N}\{\left(I(X_{i}\leq x)-F(x))(X_{i}-\bar{X}\right)\}/(N-1)$,

$S_{14}=\sum_{i=1}^{N}\{\left(I(Y_{i}\leq y)-F(y))(Z_{i}-\bar{Z}\right)\}/(N-1)$,

$S_{24}=\sum_{i=1}^{N}\{\left(I(X_{i}\leq x)-F(x))(Z_{i}-\bar{Z}\right)\}/(N-1)$.

In addition, let $R_{1.23}^{2}=(R_{12}^{2}+R_{13}^{2}-2R_{12}R_{13}R_{23})/(1-R_{23}^{2})$ be the multiple correlation coefficient of *I*(*Y*≤*y*) on *I*(*X*≤*x*) and *X*, and let $R_{1.24}^{2}=(R_{12}^{2}+R_{14}^{2}-2R_{12}R_{14}R_{24})/(1-R_{24}^{2})$ be the multiple correlation coefficient of *I*(*Y*≤*y*) on *I*(*X*≤*x*) and *Z*, under simple random sampling.

## 3 Adapted estimators in simple random sampling

In this section, some estimators of finite population mean are adapted for estimating the finite CDF under simple random sampling. The biases and MSEs of these adapted estimators are derived under the first order of approximation.

1. The traditional unbiased estimator of *F*(*y*) is

$${\hat{F}}_{1}(y)=\frac{1}{n}\sum_{i=1}^{n}I(Y_{i}\leq y).$$ (1)

The variance of ${\hat{F}}_{1}(y)$ is

$$Var({\hat{F}}_{1}(y))=F^{2}(y)V_{2000}.$$ (2)

2. Cochran [31] adapted ratio estimator of *F*(*y*) is

$${\hat{F}}_{2}(y)=\hat{F}(y)\left(\frac{F(x)}{\hat{F}(x)}\right).$$ (3)

The bias and MSE of ${\hat{F}}_{2}(y)$, to the first order of approximation, are

$$Bias({\hat{F}}_{2}(y))≅F(y)(V_{0200}-V_{1100}),$$

$$MSE({\hat{F}}_{2}(y))≅F^{2}(y)(V_{2000}+V_{0200}-2V_{1100}).$$ (4)

If *R*_12_>*C*_2_/(2*C*_1_), then ${\hat{F}}_{2}(y)$ is better than ${\hat{F}}_{1}(y)$ in terms of MSE.

3. Murthy [32] adapted product estimator of *F*(*y*) is

$${\hat{F}}_{3}(y)=\hat{F}(y)\left(\frac{\hat{F}(x)}{F(x)}\right).$$ (5)

The bias and MSE of ${\hat{F}}_{3}(y)$, to the first order of approximation, are

$$Bias({\hat{F}}_{3}(y))=F(y)V_{1100},$$

$$MSE({\hat{F}}_{3}(y))≅F^{2}(y)(V_{2000}+V_{0200}+2V_{1100}).$$ (6)

If −*C*_2_/(2*C*_1_)>*R*_12_, then ${\hat{F}}_{3}(y)$ is better than ${\hat{F}}_{1}(y)$ in terms of MSE.

4. The adapted difference estimator of *F*(*y*) is

$${\hat{F}}_{4}(y)=\hat{F}(y)+k\left(F(x)-\hat{F}(x)\right).$$ (7)

where *k* is an unknown constant. Here, ${\hat{F}}_{4}(y)$ is an unbiased estimator of *F*(*y*). The minimum variance of ${\hat{F}}_{4}(y)$ at the optimum value *k*_(opt)_ = (*F*(*y*)*V*_1100_)/(*F*(*x*)*V*_0200_) is

$$Var_{min}({\hat{F}}_{4}(y))=\frac{F^{2}(y)(V_{2000}V_{0200}-V_{1100}^{2})}{V_{0200}}.$$ (8)

Here, (8) may be written as

$$Var_{min}({\hat{F}}_{4}(y))=F^{2}(y)V_{2000}(1-R_{12}^{2}).$$ (9)

5. Rao [4] adapted difference-type estimator of *F*(*y*) is

$${\hat{F}}_{5}(y)=k_{1}\hat{F}(y)+k_{2}(F(x)-\hat{F}(x)),$$ (10)

where *k*_1_ and *k*_2_ are unknown constants. The bias and MSE of ${\hat{F}}_{5}(y)$, to the first order of approximation, are

$$Bias({\hat{F}}_{5}(y))=F(y)(k_{1}-1),$$

$$MSE({\hat{F}}_{5}(y))≅F^{2}(y)-2k_{1}F^{2}(y)+k_{1}^{2}F^{2}(y)+k_{1}^{2}F^{2}(y)V_{2000}$$

$$-2k_{1}k_{2}F(y)F(x)V_{1100}+k_{2}^{2}F^{2}(x)V_{0200}.$$ (11)

The optimum values of *k*_1_ and *k*_2_, determined by minimizing (11), are

$$k_{1(opt)}=\frac{V_{0200}}{\left(V_{0200}V_{2000}-V_{1100}^{2}+V_{0200}\right)}$$

$$k_{2(opt)}=\frac{F(y)V_{1100}}{F(x)(V_{2000}V_{0200}-V_{1100}^{2}+V_{0200})}.$$

The minimum MSE of ${\hat{F}}_{5}(y)$ at the optimum values of *k*_1_ and *k*_2_ is

$$MSE_{min}({\hat{F}}_{5}(y))=\frac{F^{2}(y)(V_{2000}V_{0200}-V_{1100}^{2})}{\left(V_{2000}V_{0200}-V_{1100}^{2}+V_{0200}\right)}.$$ (12)

Here, (12) may be written as

$$MSE_{min}({\hat{F}}_{5}(y))=\frac{F^{2}(y)V_{2000}(1-R_{12}^{2})}{1+V_{2000}(1-R_{12}^{2})}.$$ (13)

6. Singh et al. [33] adapted generalized ratio-type exponential estimator of *F*(*y*) is

$${\hat{F}}_{6}(y)=\hat{F}(y)exp\left(\frac{a(F(x)-\hat{F}(x))}{a(F(x)+\hat{F}(x))+2b}\right),$$ (14)

where *a* and *b* are known constants. The bias and MSE of ${\hat{F}}_{6}(y)$, to the first order of approximation, are

$$Bias({\hat{F}}_{6}(y))≅F(y)\left(\frac{3}{8}\theta^{2}V_{0200}-\frac{1}{2}\theta V_{1100}\right),$$

$$MSE({\hat{F}}_{6}(y))≅\frac{F^{2}(y)}{4}(4V_{2000}+\theta^{2}V_{0200}-4\theta V_{1100}),$$ (15)

where *θ* = *aF*(*x*)/(*aF*(*x*)+*b*).

7. Grover and Kaur [11] adapted generalized class of ratio-type exponential estimator of *F*(*y*) is

$${\hat{F}}_{7}(y)=\{k_{3}\hat{F}(y)+k_{4}(F(x)-\hat{F}(x))\}exp\left(\frac{a(F(x)-\hat{F}(x))}{a(F(x)+\hat{F}(x))+2b}\right),$$ (16)

where *k*_3_ and *k*_4_ are unknown constants. The bias and MSE of ${\hat{F}}_{7}(y)$, to the first order of approximation, are

$$Bias({\hat{F}}_{7}(y))≅F(y)(k_{3}-1)+\frac{3}{8}\theta^{2}k_{3}F(y)+\frac{1}{2}\theta k_{4}F(x)V_{0200}-\frac{1}{2}\theta F(y)V_{1100},$$

$$MSE({\hat{F}}_{7}(y))≅k_{4}^{2}F^{2}(x)V_{0200}+k_{3}^{2}F^{2}(y)V_{2000}+2\theta k_{3}k_{4}F(y)F(x)V_{0200}$$

$$-2k_{3}k_{4}F(y)F(x)V_{1100}+F^{2}(y)-2k_{3}F^{2}(y)+\theta k_{3}^{2}F^{2}(y)$$

$$+k_{3}F^{2}(y)V_{1100}-\theta k_{4}F(y)F(x)V_{0200}-2\theta k_{3}^{2}F^{2}(y)V_{1100}$$

$$-\frac{3}{4}\theta^{2}k_{3}F^{2}(y)V_{0200}+\theta^{2}k_{3}^{2}F^{2}(y)V_{0200}$$ (17)

The optimum values of *k*_3_ and *k*_4_, determined by minimizing (17), are

$$k_{3(opt)}=\frac{V_{0200}(\theta^{2}V_{0200}-8)}{8(-V_{2000}V_{0200}+V_{1100}^{2}-V_{0200})},$$

$$k_{4(opt)}=\frac{F(y)(\theta^{3}V_{0200}^{2}-\theta^{2}V_{0200}V_{1100}+4\theta V_{2000}V_{0200}-4\theta V_{1100}^{2}-4\theta V_{0200}+8V_{1100})}{8F(x)(V_{2000}V_{0200}-V_{1100}^{2}+V_{0200})}.$$

The simplified minimum MSE of ${\hat{F}}_{7}(y)$ at the optimum values of *k*_3_ and *k*_4_ is

$$MSE_{min}({\hat{F}}_{7}(y))≅\frac{F^{2}(y)}{64}\left(64-16\theta^{2}V_{0200}-\frac{V_{0200}(-8+\theta^{2}V_{0200}{)}^{2}}{V_{0200}(1+V_{2000})-V_{1100}^{2}}\right).$$ (18)

Here, (18) may be written as

$$MSE_{min}({\hat{F}}_{7}(y))≅Var_{min}({\hat{F}}_{4}(y))-\frac{F^{2}(y){\left(\theta^{2}V_{0200}^{2}-8V_{1100}^{2}+8V_{0200}V_{2000}\right)}^{2}}{64V_{0200}^{2}\{1+V_{2000}(1-R_{12}^{2})\}},$$ (19)

which shows that ${\hat{F}}_{7}(y)$ is more precise than ${\hat{F}}_{4}(y)$.

## 4 Proposed estimators in simple random sampling

The precision of an estimator increases by using suitable auxiliary information at the estimation stage. In previous studies, the sample distribution function of the auxiliary variable was used to improve the efficiencies of the existing distribution function estimators. In a recent study, Haq et al. [16] suggested using ranks of the auxiliary variable as an additional auxiliary variable to increase the precision of an estimator of the population mean. On similar lines, we use additional auxiliary information on sample means of the auxiliary and ranked-auxiliary variables along with the sample distribution function estimators of *F*(*y*) and *F*(*x*) to estimate the finite CDF. For this purpose, we propose two families of estimators for estimating *F*(*y*).

### 4.1 First proposed family of estimators

For the first family of estimators, the sample mean of the auxiliary variable is used as an additional auxiliary variable; whilst in the second family of estimators, the sample mean of the ranked auxiliary variable is used as an additional auxiliary variable.

On the lines of ${\hat{F}}_{5}(y)$ and ${\hat{F}}_{6}(y)$, first proposed family of estimators for estimating *F*(*y*) is given by

$${\hat{F}}_{8}(y)=\left\{k_{5}\hat{F}(y)+k_{6}(\frac{F(x)-\hat{F}(x)}{F(x)})+k_{7}(\frac{\bar{X}-\hat{\bar{X}}}{\bar{X}})\right\}exp\left(\frac{a(F(x)-\hat{F}(x))}{a(F(x)+\hat{F}(x))+2b}\right),$$ (20)

where *k*_5_, *k*_6_ and *k*_7_ are unknown constants, *a*(≠0) and *b* are either two real numbers or function of known population parameters of *I*(*X*≤*x*), like *R*_12_, *β*_2_ (coefficient of kurtosis), *C*_2_, etc.

The estimator ${\hat{F}}_{8}(y)$ can also be written as

$${\hat{F}}_{8}(y)=\{k_{5}F(y)(1+e_{1})-k_{6}e_{2}-k_{7}e_{3}\}\left(1-\frac{1}{2}\theta e_{2}+\frac{3}{8}\theta^{2}e_{2}^{2}+\cdots \right).$$ (21)

Simplifying (21) and keeping terms only up to the second power of *e*_*i*_’s, we can write

$$({\hat{F}}_{8}(y)-F(y))=-F(y)+k_{5}F(y)+k_{5}F(y)e_{1}-\frac{1}{2}\theta k_{5}F(y)e_{2}-k_{6}e_{2}-k_{7}e_{3}$$

$$+\frac{3}{8}\theta^{2}k_{5}F(y)e_{2}^{2}+\frac{1}{2}\theta k_{6}e_{2}^{2}-\frac{1}{2}\theta k_{5}F(y)e_{1}e_{2}+\frac{1}{2}\theta k_{7}e_{2}e_{3}.$$ (22)

The bias and MSE of ${\hat{F}}_{8}(y)$, to the first order of approximation, are

$$Bias({\hat{F}}_{8}(y))≅F(y)(k_{5}-1)+\frac{3}{8}\theta^{2}k_{5}F(y)V_{0200}+\frac{1}{2}\theta k_{6}V_{0200}-\frac{1}{2}\theta k_{5}F(y)V_{1100}+\frac{1}{2}\theta k_{7}V_{0110},$$

$$MSE({\hat{F}}_{8}(y))≅F^{2}(y)(k_{5}-1{)}^{2}+k_{5}^{2}F^{2}(y)V_{2000}+k_{6}^{2}V_{0200}+k_{7}^{2}V_{0020}+\theta^{2}k_{5}^{2}F^{2}(y)V_{0200}$$

$$-\theta k_{6}F(y)V_{0200}+2\theta k_{5}k_{6}F(y)V_{0200}-\frac{3}{4}\theta^{2}k_{5}F^{2}(y)V_{0200}+\theta k_{5}F^{2}(y)V_{1100}$$

$$-2\theta k_{5}^{2}F^{2}(y)V_{1100}-2k_{5}k_{6}F(y)V_{1100}-2k_{5}k_{7}F(y)V_{1010}-\theta k_{7}F(y)V_{0110}$$

$$+2\theta k_{5}k_{7}F(y)V_{0110}-2k_{6}k_{7}V_{0110}.$$ (23)

The optimum values of *k*_5_, *k*_6_ and *k*_7_, determined by minimizing (23), are

$$k_{5(opt)}=\frac{8-\theta^{2}V_{0200}}{8\{1+V_{2000}(1-R_{1.23}^{2})\}},$$

$$k_{6(opt)}=\frac{F(y)[\begin{array}{ll} & \theta^{3}V_{0200}^{3/2}(R_{23}^{2}-1)+V_{2000}^{1/2}(-8+\theta^{2}V_{0200})(R_{12}-R_{23}R_{13})\\ & +4\theta V_{0200}^{1/2}(R_{23}^{2}-1)\{-1+V_{2000}(1-R_{1.23}^{2})\} \end{array}]}{8V_{0200}^{1/2}(R_{23}^{2}-1)\{-1+V_{2000}(1-R_{1.23}^{2})\}},$$

$$k_{7(opt)}=\frac{F(y)V_{2000}^{1/2}(8-\theta^{2}V_{0200})(R_{12}-R_{23}R_{13})}{8V_{0200}^{1/2}(R_{23}^{2}-1)\{-1+V_{2000}(1-R_{1.23}^{2})\}}.$$

The simplified minimum MSE of ${\hat{F}}_{8}(y)$ at the optimum values of *k*_5_, *k*_6_ and *k*_7_ is

$$MSE_{min}({\hat{F}}_{8}(y))≅\frac{F^{2}(y)\{64V_{2000}(1-R_{1.23}^{2})-\theta^{4}V_{0200}^{2}-16\theta^{2}V_{0200}V_{2000}(1-R_{1.23}^{2})\}}{64\{1+V_{2000}(1-R_{1.23}^{2})\}},$$ (24)

where $R_{1.23}^{2}=\left(\frac{V_{1100}^{2}V_{0020}+V_{1010}^{2}V_{0200}-2V_{1010}V_{1100}V_{0110}}{V_{2000}(V_{0200}V_{0020}-V_{0110}^{2})}\right)$.

Here, (24) may be written as

$$MSE_{min}({\hat{F}}_{8}(y))≅Var_{min}({\hat{F}}_{4}(y))-H_{1}-H_{2},$$ (25)

where

$$H_{1}=\frac{F^{2}(y){\left(\theta^{2}V_{0200}^{2}-8V_{1100}^{2}+8V_{0200}V_{2000}\right)}^{2}}{64V_{0200}^{2}\{1+V_{2000}(1-R_{12}^{2})\}}and$$

$$H_{2}=\frac{F^{2}(y){\left(\theta^{2}V_{0200}-8\right)}^{2}{\left(V_{0200}V_{1010}-V_{0110}V_{1100}\right)}^{2}}{64V_{0200}^{2}V_{0020}(1-R_{23}^{2})\{1+V_{2000}(1-R_{12}^{2})\}\{1+V_{2000}(1-R_{1.23}^{2})\}}.$$

It can be seen that ${\hat{F}}_{8}(y)$ is more precise than ${\hat{F}}_{4}(y)$.

### 4.2 Second proposed family of estimators

On similar lines, second proposed family of estimators for estimating *F*(*y*) is given by

$${\hat{F}}_{9}(y)=\left\{k_{8}\hat{F}(y)+k_{9}(\frac{F(x)-\hat{F}(x)}{F(x)})+k_{10}(\frac{\bar{Z}-\hat{\bar{Z}}}{\bar{Z}})\right\}exp\left(\frac{a(F(x)-\hat{F}(x))}{a(F(x)-\hat{F}(x))+2b}\right),$$ (26)

where *k*_8_, *k*_9_ and *k*_10_ are unknown constants, *a*(≠0) and *b* are either two real numbers or functions of known population parameters of *I*(*X*≤*x*), like *R*_12_, *β*_2_ (coefficient of kurtosis), *C*_2_, etc. The estimator ${\hat{F}}_{9}(y)$ can also be written as

$${\hat{F}}_{9}(y)=\{k_{8}F(y)(1+e_{1})-k_{9}e_{2}-k_{10}e_{4}\}\left(1-\frac{1}{2}\theta e_{2}+\frac{3}{8}\theta^{2}e_{2}^{2}+\cdots \right).$$ (27)

Simplifying (27) and keeping terms only up to the second power of *e*_*i*_’s, we can write

$$({\hat{F}}_{9}(y)-F(y))=-F(y)+k_{8}F(y)+k_{8}F(y)e_{1}-\frac{1}{2}\theta k_{8}F(y)e_{2}-k_{9}e_{2}-k_{10}e_{4}$$

$$+\frac{3}{8}\theta^{2}k_{8}F(y)e_{2}^{2}+\frac{1}{2}\theta k_{9}e_{2}^{2}-\frac{1}{2}\theta k_{8}F(y)e_{1}e_{2}+\frac{1}{2}\theta k_{10}e_{2}e_{4}.$$ (28)

The bias and MSE of ${\hat{F}}_{9}(y)$, to the first order of approximation, are

$$Bias({\hat{F}}_{9}(y))≅F(y)(k_{8}-1)+\frac{3}{8}\theta^{2}k_{8}F(y)V_{0200}+\frac{1}{2}\theta k_{9}V_{0200}-\frac{1}{2}\theta k_{8}F(y)V_{1100}+\frac{1}{2}\theta k_{9}V_{0101},$$

$$MSE({\hat{F}}_{9}(y))≅F^{2}(y)(k_{8}-1{)}^{2}+k_{8}^{2}F^{2}(y)V_{2000}+k_{9}^{2}V_{0200}+k_{10}^{2}V_{0002}+\theta^{2}k_{8}^{2}F^{2}(y)V_{0200}$$

$$-\theta k_{9}F(y)V_{0200}+2\theta k_{8}k_{9}F(y)V_{0200}-\frac{3}{4}\theta^{2}k_{8}F^{2}(y)V_{0200}+\theta k_{8}F^{2}(y)V_{1100}$$

$$-2\theta k_{8}^{2}F^{2}(y)V_{1100}-2k_{8}k_{9}F(y)V_{1100}-2k_{8}k_{10}F(y)V_{1001}-\theta k_{10}F(y)V_{0101}$$

$$+2\theta k_{8}k_{10}F(y)V_{0101}-2k_{9}k_{10}V_{0101}.$$ (29)

The optimum values of *k*_8_, *k*_9_ and *k*_10_, determined by minimizing (29), are

$$k_{8(opt)}=\frac{8-\theta^{2}V_{0200}}{8\{1+V_{2000}(1-R_{1.24}^{2})\}},$$

$$k_{9(opt)}=\frac{F(y)[\begin{array}{ll} & \theta^{3}V_{0200}^{3/2}(R_{24}^{2}-1)+V_{2000}^{1/2}(-8+\theta^{2}V_{0200})(R_{12}-R_{24}R_{14})\\ & +4\theta V_{0200}^{1/2}(R_{24}^{2}-1)\{-1+V_{2000}(1-R_{1.24}^{2})\} \end{array}]}{8V_{0200}^{1/2}(R_{24}^{2}-1)\{-1+V_{2000}(1-R_{1.24}^{2})\}},$$

$$k_{10(opt)}=\frac{F(y)V_{2000}^{1/2}(8-\theta^{2}V_{0200})(R_{12}-R_{24}R_{14})}{8V_{0200}^{1/2}(R_{24}^{2}-1)\{-1+V_{2000}(1-R_{1.24}^{2})\}}.$$

The simplified minimum MSE of ${\hat{F}}_{9}(y)$ at the optimum values of *k*_8_, *k*_9_ and *k*_10_ is

$$MSE_{min}({\hat{F}}_{9}(y))≅\frac{F^{2}(y)\{64V_{2000}(1-R_{1.24}^{2})-\theta^{4}V_{0200}^{2}-16\theta V_{0200}V_{2000}(1-R_{1.24}^{2})\}}{64\{1+V_{2000}(1-R_{1.24}^{2})\}},$$ (30)

where $R_{1.24}^{2}=\left(\frac{V_{1100}^{2}V_{0002}+V_{1001}^{2}V_{0200}-2V_{1001}V_{1100}V_{0101}}{V_{2000}(V_{0200}V_{0002}-V_{0101}^{2})}\right)$.

Here, (30) may be written as

$$MSE_{min}({\hat{F}}_{9}(y))≅Var_{min}({\hat{F}}_{4}(y))-H_{1}-H_{3},$$ (31)

where

$$H_{1}=\frac{F^{2}(y){\left(\theta^{2}V_{0200}^{2}-8V_{1100}^{2}+8V_{0200}\right)}^{2}}{64V_{0200}^{2}\{1+V_{2000}(1-R_{12}^{2})\}}and$$

$$H_{3}=\frac{F^{2}(y){\left(\theta^{2}V_{0200}-8\right)}^{2}{\left(V_{0200}V_{1001}-V_{0101}V_{1100}\right)}^{2}}{64V_{0200}^{2}V_{0002}(1-R_{24}^{2})\{1+V_{2000}(1-R_{12}^{2})\}\{1+V_{2000}(1-R_{1.24}^{2})\}}.$$

It is clear that ${\hat{F}}_{9}(y)$ is more precise than ${\hat{F}}_{4}(y)$.

In Table 1, we put some members of the Singh et al. [33], Grover and Kaur [11], and proposed families of estimators with selected choices of *a* and *b*.

**Table 1:** Some members of the adapted and proposed distribution function estimators.

*a*	b*b*	${\hat{F}}_{6}(y)$	${\hat{F}}_{7}(y)$	${\hat{F}}_{8}(y)$	${\hat{F}}_{9}(y)$
1	C*C*_2_	${\hat{F}}_{6}^{\left(1\right)}(y)$	${\hat{F}}_{7}^{\left(1\right)}(y)$	${\hat{F}}_{8}^{\left(1\right)}(y)$	${\hat{F}}_{9}^{\left(1\right)}(y)$
1	*β*_2_	${\hat{F}}_{6}^{\left(1\right)}(y)$	${\hat{F}}_{7}^{\left(1\right)}(y)$	${\hat{F}}_{8}^{\left(1\right)}(y)$	${\hat{F}}_{9}^{\left(1\right)}(y)$
*β*_2_	*C*_2_	${\hat{F}}_{6}^{\left(3\right)}(y)$	${\hat{F}}_{7}^{\left(3\right)}(y)$	${\hat{F}}_{8}^{\left(3\right)}(y)$	${\hat{F}}_{9}^{\left(3\right)}(y)$
*C*_2_	*β*_2_	${\hat{F}}_{6}^{\left(4\right)}(y)$	${\hat{F}}_{7}^{\left(4\right)}(y)$	${\hat{F}}_{8}^{\left(4\right)}(y)$	${\hat{F}}_{9}^{\left(4\right)}(y)$
1	*R*_12_	${\hat{F}}_{6}^{\left(5\right)}(y)$	${\hat{F}}_{7}^{\left(5\right)}(y)$	${\hat{F}}_{8}^{\left(5\right)}(y)$	${\hat{F}}_{9}^{\left(5\right)}(y)$
C*C*_2_	*R*_12_	${\hat{F}}_{6}^{\left(6\right)}(y)$	${\hat{F}}_{7}^{\left(6\right)}(y)$	${\hat{F}}_{8}^{\left(6\right)}(y)$	${\hat{F}}_{9}^{\left(6\right)}(y)$
*R*_12_	*C*_2_	${\hat{F}}_{6}^{\left(7\right)}(y)$	${\hat{F}}_{7}^{\left(7\right)}(y)$	${\hat{F}}_{8}^{\left(7\right)}(y)$	${\hat{F}}_{9}^{\left(7\right)}(y)$
*β*_2_	*R*_12_	${\hat{F}}_{6}^{\left(8\right)}(y)$	${\hat{F}}_{7}^{\left(8\right)}(y)$	${\hat{F}}_{8}^{\left(8\right)}(y)$	${\hat{F}}_{9}^{\left(8\right)}(y)$
*R*_12_	*β*_2_	${\hat{F}}_{6}^{\left(9\right)}(y)$	${\hat{F}}_{7}^{\left(9\right)}(y)$	${\hat{F}}_{8}^{\left(9\right)}(y)$	${\hat{F}}_{9}^{\left(9\right)}(y)$
1	*NF*(*x*)	${\hat{F}}_{6}^{\left(10\right)}(y)$	${\hat{F}}_{7}^{\left(10\right)}(y)$	${\hat{F}}_{8}^{\left(1\right)}(y)$	${\hat{F}}_{9}^{\left(10\right)}(y)$

## 5 Efficiency comparisons in simple random sampling

In this section, the adapted and proposed estimators of *F*(*y*) are compared in terms of the minimum MSEs. [(i)]

1. From (2) and (25),

$$MSE_{min}({\hat{F}}_{8}(y))<Var({\hat{F}}_{1}(y))if$$

$$F^{2}(y)V_{2000}R_{12}^{2}+H_{1}+H_{2}>0.$$

2. From (4) and (25),

$$MSE_{min}({\hat{F}}_{8}(y))<MSE({\hat{F}}_{2}(y))if$$

$$\frac{F^{2}(y)}{V_{2000}}{\left(V_{0200}-V_{1100}\right)}^{2}+H_{1}+H_{2}>0.$$

3. From (6) and (25),

$$MSE_{min}({\hat{F}}_{8}(y))<MSE({\hat{F}}_{3}(y))if$$

$$\frac{F^{2}(y)}{V_{2000}}{\left(V_{0200}+V_{1100}\right)}^{2}+H_{1}+H_{2}>0.$$

4. From (9) and (25),

$$MSE_{min}({\hat{F}}_{8}(y))<MSE_{min}({\hat{F}}_{4}(y))if$$

$$H_{1}+H_{2}>0.$$

5. From (13) and (25),

$$MSE_{min}({\hat{F}}_{8}(y))<MSE_{min}({\hat{F}}_{5}(y))if$$

$$\frac{F^{2}(y)\theta^{2}V_{0200}\{\theta^{2}V_{0200}+16V_{2000}(1-R_{12}^{2})\}}{64\{1+V_{2000}(1-R_{12}^{2})\}}+H_{2}>0.$$

6. From (15) and (25),

$$MSE_{min}({\hat{F}}_{8}(y))<MSE({\hat{F}}_{6}(y))if$$

$$\frac{F^{2}(y)}{V_{2000}}{\left(\frac{\theta V_{0200}}{2}-V_{1100}\right)}^{2}+H_{1}+H_{2}>0.$$

7. From (19) and (25),

$$MSE_{min}({\hat{F}}_{8}(y))<MSE_{min}({\hat{F}}_{7}(y))if$$

$$H_{2}>0.$$

8. From (2) and (31),

$$MSE_{min}({\hat{F}}_{9}(y))<Var({\hat{F}}_{1}(y))if$$

$$\frac{1}{V_{0200}}F^{2}(y)V_{1100}^{2}+H_{1}+H_{3}>0.$$

9. From (4) and (31),

$$MSE_{min}({\hat{F}}_{9}(y))<MSE({\hat{F}}_{2}(y))if$$

$$\frac{F^{2}(y)}{V_{2000}}{\left(V_{0200}-V_{1100}\right)}^{2}+H_{1}+H_{3}>0.$$

10. From (6) and (31),

$$MSE_{min}({\hat{F}}_{9}(y))<MSE({\hat{F}}_{3}(y))if$$

$$\frac{F^{2}(y)}{V_{2000}}{\left(V_{0200}+V_{1100}\right)}^{2}+H_{1}+H_{3}>0.$$

11. From (9) and (31),

$$MSE_{min}({\hat{F}}_{9}(y))<MSE_{min}({\hat{F}}_{4}(y))if$$

$$H_{1}+H_{3}>0.$$

12. From (13) and (31),

$$MSE_{min}({\hat{F}}_{9}(y))<MSE_{min}({\hat{F}}_{5}(y))if$$

$$\frac{F^{2}(y)\theta^{2}V_{0200}\{\theta^{2}V_{0200}+16V_{2000}(1-R_{12}^{2})\}}{64\{1+V_{2000}(1-R_{12}^{2})\}}+H_{3}>0.$$

13. From (15) and (31),

$$MSE_{min}({\hat{F}}_{9}(y))<MSE_{min}({\hat{F}}_{6}(y))if$$

$$\frac{F^{2}(y)}{V_{2000}}{\left(\frac{\theta V_{0200}}{2}-V_{1100}\right)}^{2}+H_{1}+H_{3}>0.$$

14. From (19) and (31),

$$MSE_{min}({\hat{F}}_{9}(y))<MSE_{min}({\hat{F}}_{7}(y))if$$

$$H_{3}>0.$$

The proposed families of estimators are always more precise than the adapted estimators as the above conditions (i)–(xiv) are always true.

## 6 Empirical study in simple random sampling

In this section, we conduct a numerical study to investigate the performances of the adapted and CDF estimators. For this purpose, five populations are considered. The summary statistics of these populations are reported in Table 2. The percentage relative efficiency (PRE) of an estimator ${\hat{F}}_{i}(y)$ with respect to ${\hat{F}}_{1}(y)$ is

$$PRE({\hat{F}}_{i}(y),{\hat{F}}_{1}(y))=\frac{Var({\hat{F}}_{1}(y))}{MSE_{min}({\hat{F}}_{i}(y))}\times 100,$$

where *i* = 2,3,…,9.

**Table 2:** Summary statistics for Populations I to V.

Population	I	II	III	IV	V
*N*	30	50	80	120	923
*n*	5	5	10	20	180
*λ*	0.16667	0.18	0.0875	0.04167	0.00447
$\bar{X}$	67.267	78.29	285.125	33.535	11440.5
*C*_3_	0.13725	0.27229	0.94846	3.32354	1.86453
$\bar{R}$	15.5	25.5	40.5	60.5	462
*C*_4_	0.56695	0.57166	0.57377	0.57493	0.57703
*m*_2(*y*)_	387	831	5105	9.25	171
*m*_2(*x*)_	66.5	75.35	148	8.6	4123
$R_{1.23}^{2}$	0.5751	0.06242	0.90458	0.93453	0.72087
$R_{1.24}^{2}$	0.5783	0.0403	0.907	0.93725	0.75269
*F*(*y*)	0.5	0.5	0.5	0.5	0.50163
*C*_1_	1.01709	1.01015	1.00631	1.00419	0.99729
*F*(*x*)	0.5	0.5	0.5	0.5	0.50054
*C*_2_	1.01709	1.01015	1.00631	1.00419	0.99946
*R*_12_	−0.73333	−0.12000	0.95	0.96667	0.84616
*R*_13_	0.71975	0.22925	−0.71920	- 0.26749	-0.44165
*R*_23_	−0.83720	−0.78936	−0.72395	−0.26760	−0.44809
*R*_14_	0.73622	0.18435	−0.85636	−0.86368	−0.82860
*R*_24_	−0.86727	−0.86630	−0.86610	−0.86609	−0.86603
*β*_2_	1	1	1	1	1

The PREs of distribution function estimators, computed from five populations, are given in Tables 7–11.

**Population I** (Source: Singh, [6])

*Y*: Duration of sleep (in minutes) and

*X*: Age of old persons.

**Population II** (Source: Gujarati, [34])

*Y*: The eggs produced in 1990 (millions) and

*X*: The price per dozen (cents) in 1990.

**Population III** (Source: Murthy, [1])

*Y*: The output of the factory and

*X*: The number of workers.

**Population IV** (Source: Sarndal, [35])

*Y*: Population in 1983 (in million) and

*X*: Population in 1980 (in million).

**Population V** (Source: Koyuncu and Kadilar, [36])

*Y*: Number of teachers and

*X*: number of students.

From the numerical results, presented in Tables 3–7, it is observed that the PREs of all families of estimators change with the choice of *a* or *b*. It is further noted that the proposed families of estimators are more precise than the adapted distribution function estimators of Cochran [31], Murthy [32], Rao [4], Singh et al. [33] and Grover and Kaur [11], in terms of PRE. It can be seen that, for data sets I, III, IV and V, the second proposed family of the estimators perform better than the first proposed family of estimators, and for data set-II, it is also observed that the first proposed family of the estimators perform better than the second proposed family of estimators.

**Table 3:** PREs of distribution function estimators using Population I.

Estimator	Value	Estimator	Value	Estimator	Value	Estimator	Value	Estimator	Value
${\hat{F}}_{1}(y)$	100	${\hat{F}}_{6}^{\left(1\right)}(y)$	78.81	${\hat{F}}_{7}^{\left(1\right)}(y)$	234.70	${\hat{F}}_{8}^{\left(1\right)}(y)$	253.84	${\hat{F}}_{9}^{\left(1\right)}(y)$	255.59
${\hat{F}}_{2}(y)$	28.84	${\hat{F}}_{6}^{\left(2\right)}(y)$	78.60	${\hat{F}}_{7}^{\left(2\right)}(y)$	234.72	${\hat{F}}_{8}^{\left(2\right)}(y)$	253.87	${\hat{F}}_{9}^{\left(2\right)}(y)$	255.62
${\hat{F}}_{3}(y)$	187.50	${\hat{F}}_{6}^{\left(3\right)}(y)$	78.81	${\hat{F}}_{7}^{\left(3\right)}(y)$	234.70	${\hat{F}}_{8}^{\left(3\right)}(y)$	253.84	${\hat{F}}_{9}^{\left(3\right)}(y)$	255.59
${\hat{F}}_{4}(y)$	216.34	${\hat{F}}_{6}^{\left(4\right)}(y)$	78.39	${\hat{F}}_{7}^{\left(4\right)}(y)$	234.75	${\hat{F}}_{8}^{\left(4\right)}(y)$	253.90	${\hat{F}}_{9}^{\left(4\right)}(y)$	255.65
${\hat{F}}_{5}(y)$	233.58	${\hat{F}}_{6}^{\left(5\right)}(y)$	173.45	${\hat{F}}_{7}^{\left(5\right)}(y)$	343.92	${\hat{F}}_{8}^{\left(5\right)}(y)$	377.99	${\hat{F}}_{9}^{\left(5\right)}(y)$	381.16
		${\hat{F}}_{6}^{\left(6\right)}(y)$	161.15	${\hat{F}}_{7}^{\left(6\right)}(y)$	372.72	${\hat{F}}_{8}^{\left(6\right)}(y)$	411.96	${\hat{F}}_{9}^{\left(6\right)}(y)$	415.63
		${\hat{F}}_{6}^{\left(7\right)}(y)$	150.14	${\hat{F}}_{7}^{\left(7\right)}(y)$	236.97	${\hat{F}}_{8}^{\left(7\right)}(y)$	256.31	${\hat{F}}_{9}^{\left(7\right)}(y)$	258.08
		${\hat{F}}_{6}^{\left(8\right)}(y)$	173.45	${\hat{F}}_{7}^{\left(8\right)}(y)$	343.92	${\hat{F}}_{8}^{\left(8\right)}(y)$	377.99	${\hat{F}}_{9}^{\left(8\right)}(y)$	381.16
		${\hat{F}}_{6}^{\left(9\right)}(y)$	151.69	${\hat{F}}_{7}^{\left(9\right)}(y)$	237.16	${\hat{F}}_{8}^{\left(9\right)}(y)$	256.52	${\hat{F}}_{9}^{\left(9\right)}(y)$	258.29
		${\hat{F}}_{6}^{\left(10\right)}(y)$	97.66	${\hat{F}}_{7}^{\left(10\right)}(y)$	233.59	${\hat{F}}_{8}^{\left(10\right)}(y)$	252.65	${\hat{F}}_{9}^{\left(10\right)}(y)$	254.39

**Table 4:** PREs of distribution function estimators using Population II.

Estimator	Value	Estimator	Value	Estimator	Value	Estimator	Value	Estimator	Value
${\hat{F}}_{1}(y)$	100	${\hat{F}}_{6}^{\left(1\right)}(y)$	93.70	${\hat{F}}_{7}^{\left(1\right)}(y)$	120.43	${\hat{F}}_{8}^{\left(1\right)}(y)$	125.66	${\hat{F}}_{9}^{\left(1\right)}(y)$	123.19
${\hat{F}}_{2}(y)$	44.642	${\hat{F}}_{6}^{\left(2\right)}(y)$	93.65	${\hat{F}}_{7}^{\left(2\right)}(y)$	120.44	${\hat{F}}_{8}^{\left(2\right)}(y)$	125.67	${\hat{F}}_{9}^{\left(2\right)}(y)$	123.19
${\hat{F}}_{3}(y)$	56.810	${\hat{F}}_{6}^{\left(3\right)}(y)$	93.70	${\hat{F}}_{7}^{\left(3\right)}(y)$	120.43	${\hat{F}}_{8}^{\left(3\right)}(y)$	125.66	${\hat{F}}_{9}^{\left(3\right)}(y)$	123.19
${\hat{F}}_{4}(y)$	101.46	${\hat{F}}_{6}^{\left(4\right)}(y)$	93.59	${\hat{F}}_{7}^{\left(4\right)}(y)$	120.45	${\hat{F}}_{8}^{\left(4\right)}(y)$	125.67	${\hat{F}}_{9}^{\left(4\right)}(y)$	123.20
${\hat{F}}_{5}(y)$	119.82	${\hat{F}}_{6}^{\left(5\right)}(y)$	62.86	${\hat{F}}_{7}^{\left(5\right)}(y)$	131.42	${\hat{F}}_{8}^{\left(5\right)}(y)$	137.18	${\hat{F}}_{9}^{\left(5\right)}(y)$	134.46
		${\hat{F}}_{6}^{\left(6\right)}(y)$	62.99	${\hat{F}}_{7}^{\left(6\right)}(y)$	131.33	${\hat{F}}_{8}^{\left(6\right)}(y)$	137.09	${\hat{F}}_{9}^{\left(6\right)}(y)$	134.37
		${\hat{F}}_{6}^{\left(7\right)}(y)$	100.66	${\hat{F}}_{7}^{\left(7\right)}(y)$	119.85	${\hat{F}}_{8}^{\left(7\right)}(y)$	125.04	${\hat{F}}_{9}^{\left(7\right)}(y)$	122.58
		${\hat{F}}_{6}^{\left(8\right)}(y)$	62.86	${\hat{F}}_{7}^{\left(8\right)}(y)$	131.42	${\hat{F}}_{8}^{\left(8\right)}(y)$	137.18	${\hat{F}}_{9}^{\left(8\right)}(y)$	134.46
		${\hat{F}}_{6}^{\left(9\right)}(y)$	100.66	${\hat{F}}_{7}^{\left(9\right)}(y)$	119.85	${\hat{F}}_{8}^{\left(9\right)}(y)$	125.04	${\hat{F}}_{9}^{\left(9\right)}(y)$	122.58
		${\hat{F}}_{6}^{\left(10\right)}(y)$	99.75	${\hat{F}}_{7}^{\left(10\right)}(y)$	119.83	${\hat{F}}_{8}^{\left(10\right)}(y)$	125.02	${\hat{F}}_{9}^{\left(10\right)}(y)$	122.56

**Table 5:** PREs of distribution function estimators using Population III.

Estimator	Value	Estimator	Value	Estimator	Value	Estimator	Value	Estimator	Value
${\hat{F}}_{1}(y)$	100	${\hat{F}}_{6}^{\left(1\right)}(y)$	140.40	${\hat{F}}_{7}^{\left(1\right)}(y)$	1037.21	${\hat{F}}_{8}^{\left(1\right)}(y)$	1059.61	${\hat{F}}_{9}^{\left(1\right)}(y)$	1087.07
${\hat{F}}_{2}(y)$	1000	${\hat{F}}_{6}^{\left(2\right)}(y)$	140.62	${\hat{F}}_{7}^{\left(2\right)}(y)$	1037.23	${\hat{F}}_{8}^{\left(2\right)}(y)$	1059.64	${\hat{F}}_{9}^{\left(2\right)}(y)$	1087.09
${\hat{F}}_{3}(y)$	25.64	${\hat{F}}_{6}^{\left(3\right)}(y)$	140.40	${\hat{F}}_{7}^{\left(3\right)}(y)$	1037.21	${\hat{F}}_{8}^{\left(3\right)}(y)$	1059.61	${\hat{F}}_{9}^{\left(3\right)}(y)$	1087.07
${\hat{F}}_{4}(y)$	1025.64	${\hat{F}}_{6}^{\left(4\right)}(y)$	140.84	${\hat{F}}_{7}^{\left(4\right)}(y)$	1037.26	${\hat{F}}_{8}^{\left(4\right)}(y)$	1059.66	${\hat{F}}_{9}^{\left(4\right)}(y)$	1087.12
${\hat{F}}_{5}(y)$	1034.50	${\hat{F}}_{6}^{\left(5\right)}(y)$	142.42	${\hat{F}}_{7}^{\left(5\right)}(y)$	1037.44	${\hat{F}}_{8}^{\left(5\right)}(y)$	1059.85	${\hat{F}}_{9}^{\left(5\right)}(y)$	1087.31
		${\hat{F}}_{6}^{\left(6\right)}(y)$	142.64	${\hat{F}}_{7}^{\left(6\right)}(y)$	1037.46	${\hat{F}}_{8}^{\left(6\right)}(y)$	1059.88	${\hat{F}}_{9}^{\left(6\right)}(y)$	1087.34
		${\hat{F}}_{6}^{\left(7\right)}(y)$	138.68	${\hat{F}}_{7}^{\left(7\right)}(y)$	1037.02	${\hat{F}}_{8}^{\left(7\right)}(y)$	1059.42	${\hat{F}}_{9}^{\left(7\right)}(y)$	1086.87
		${\hat{F}}_{6}^{\left(8\right)}(y)$	142.42	${\hat{F}}_{7}^{\left(8\right)}(y)$	1037.44	${\hat{F}}_{8}^{\left(8\right)}(y)$	1059.85	${\hat{F}}_{9}^{\left(8\right)}(y)$	1087.31
		${\hat{F}}_{6}^{\left(9\right)}(y)$	138.89	${\hat{F}}_{7}^{\left(9\right)}(y)$	1037.04	${\hat{F}}_{8}^{\left(9\right)}(y)$	1059.4	${\hat{F}}_{9}^{\left(9\right)}(y)$	1086.89
		${\hat{F}}_{6}^{\left(10\right)}(y)$	101.18	${\hat{F}}_{7}^{\left(10\right)}(y)$	1034.50	${\hat{F}}_{8}^{\left(10\right)}(y)$	1056.84	${\hat{F}}_{9}^{\left(10\right)}(y)$	1084.22

**Table 6:** PREs of distribution function estimators using Population IV.

Estimator	Value	Estimator	Value	Estimator	Value	Estimator	Value	Estimator	Value
${\hat{F}}_{1}(y)$	100	${\hat{F}}_{6}^{\left(1\right)}(y)$	141.58	${\hat{F}}_{7}^{\left(1\right)}(y)$	1531.59	${\hat{F}}_{8}^{\left(1\right)}(y)$	1533.54	${\hat{F}}_{9}^{\left(1\right)}(y)$	1599.81
${\hat{F}}_{2}(y)$	1500.00	${\hat{F}}_{6}^{\left(2\right)}(y)$	141.73	${\hat{F}}_{7}^{\left(2\right)}(y)$	1531.60	${\hat{F}}_{8}^{\left(2\right)}(y)$	1533.55	${\hat{F}}_{9}^{\left(2\right)}(y)$	1599.82
${\hat{F}}_{3}(y)$	25.42	${\hat{F}}_{6}^{\left(3\right)}(y)$	141.58	${\hat{F}}_{7}^{\left(3\right)}(y)$	1531.59	${\hat{F}}_{8}^{\left(3\right)}(y)$	1533.54	${\hat{F}}_{9}^{\left(3\right)}(y)$	1599.81
${\hat{F}}_{4}(y)$	1525.42	${\hat{F}}_{6}^{\left(4\right)}(y)$	141.88	${\hat{F}}_{7}^{\left(4\right)}(y)$	1531.61	${\hat{F}}_{8}^{\left(4\right)}(y)$	1533.56	${\hat{F}}_{9}^{\left(4\right)}(y)$	1599.84
${\hat{F}}_{5}(y)$	1529.62	${\hat{F}}_{6}^{\left(5\right)}(y)$	142.95	${\hat{F}}_{7}^{\left(5\right)}(y)$	1531.70	${\hat{F}}_{8}^{\left(5\right)}(y)$	1533.65	${\hat{F}}_{9}^{\left(5\right)}(y)$	1599.93
		${\hat{F}}_{6}^{\left(6\right)}(y)$	143.11	${\hat{F}}_{7}^{\left(6\right)}(y)$	1531.71	${\hat{F}}_{8}^{\left(6\right)}(y)$	1533.66	${\hat{F}}_{9}^{\left(6\right)}(y)$	1599.94
		${\hat{F}}_{6}^{\left(7\right)}(y)$	140.39	${\hat{F}}_{7}^{\left(7\right)}(y)$	1531.49	${\hat{F}}_{8}^{\left(7\right)}(y)$	1533.44	${\hat{F}}_{9}^{\left(7\right)}(y)$	1599.71
		${\hat{F}}_{6}^{\left(8\right)}(y)$	142.95	${\hat{F}}_{7}^{\left(8\right)}(y)$	1531.70	${\hat{F}}_{8}^{\left(8\right)}(y)$	1533.65	${\hat{F}}_{9}^{\left(8\right)}(y)$	1599.93
		${\hat{F}}_{6}^{\left(9\right)}(y)$	140.53	${\hat{F}}_{7}^{\left(9\right)}(y)$	1531.50	${\hat{F}}_{8}^{\left(9\right)}(y)$	1533.45	${\hat{F}}_{9}^{\left(9\right)}(y)$	1599.72
		${\hat{F}}_{6}^{\left(10\right)}(y)$	100.80	${\hat{F}}_{7}^{\left(10\right)}(y)$	1529.62	${\hat{F}}_{8}^{\left(10\right)}(y)$	1531.57	${\hat{F}}_{9}^{\left(10\right)}(y)$	1597.75

**Table 7:** PREs of distribution function estimators using Population V.

Estimator	Value	Estimator	Value	Estimator	Value	Estimator	Value	Estimator	Value
${\hat{F}}_{1}(y)$	100	${\hat{F}}_{6}^{\left(1\right)}(y)$	134.23	${\hat{F}}_{7}^{\left(1\right)}(y)$	352.57	${\hat{F}}_{8}^{\left(1\right)}(y)$	358.74	${\hat{F}}_{9}^{\left(1\right)}(y)$	404.86
${\hat{F}}_{2}(y)$	324.29	${\hat{F}}_{6}^{\left(2\right)}(y)$	134.21	${\hat{F}}_{7}^{\left(2\right)}(y)$	352.57	${\hat{F}}_{8}^{\left(2\right)}(y)$	358.74	${\hat{F}}_{9}^{\left(2\right)}(y)$	404.86
${\hat{F}}_{3}(y)$	27.02	${\hat{F}}_{6}^{\left(3\right)}(y)$	134.23	${\hat{F}}_{7}^{\left(3\right)}(y)$	352.57	${\hat{F}}_{8}^{\left(3\right)}(y)$	358.74	${\hat{F}}_{9}^{\left(3\right)}(y)$	404.86
${\hat{F}}_{4}(y)$	352.08	${\hat{F}}_{6}^{\left(4\right)}(y)$	134.20	${\hat{F}}_{7}^{\left(4\right)}(y)$	352.57	${\hat{F}}_{8}^{\left(4\right)}(y)$	358.74	${\hat{F}}_{9}^{\left(4\right)}(y)$	404.86
${\hat{F}}_{5}(y)$	352.53	${\hat{F}}_{6}^{\left(5\right)}(y)$	38.98	${\hat{F}}_{7}^{\left(5\right)}(y)$	352.58	${\hat{F}}_{8}^{\left(5\right)}(y)$	358.75	${\hat{F}}_{9}^{\left(5\right)}(y)$	404.87
		${\hat{F}}_{6}^{\left(6\right)}(y)$	138.96	${\hat{F}}_{7}^{\left(6\right)}(y)$	352.58	${\hat{F}}_{8}^{\left(6\right)}(y)$	358.75	${\hat{F}}_{9}^{\left(6\right)}(y)$	404.87
		${\hat{F}}_{6}^{\left(7\right)}(y)$	129.89	${\hat{F}}_{7}^{\left(7\right)}(y)$	352.56	${\hat{F}}_{8}^{\left(7\right)}(y)$	358.73	${\hat{F}}_{9}^{\left(7\right)}(y)$	404.85
		${\hat{F}}_{6}^{\left(8\right)}(y)$	138.98	${\hat{F}}_{7}^{\left(8\right)}(y)$	352.58	${\hat{F}}_{8}^{\left(8\right)}(y)$	358.75	${\hat{F}}_{9}^{\left(8\right)}(y)$	404.87
		${\hat{F}}_{6}^{\left(9\right)}(y)$	129.88	${\hat{F}}_{7}^{\left(9\right)}(y)$	352.56	${\hat{F}}_{8}^{\left(9\right)}(y)$	358.73	${\hat{F}}_{9}^{\left(9\right)}(y)$	404.85
		${\hat{F}}_{6}^{\left(10\right)}(y)$	100.09	${\hat{F}}_{7}^{\left(10\right)}(y)$	352.53	${\hat{F}}_{8}^{\left(10\right)}(y)$	358.69	${\hat{F}}_{9}^{\left(10\right)}(y)$	404.80

Here we take five data sets for numerical illustration. We selected different sample sizes from these populations and, then, we used simple random sampling. The MSEs (minimum) of the proposed families of estimators are pointed out in Eqs 25 and 31. Finally, the adapted estimators and proposed families of estimators were compared with each other with respect to their PRE values. These results are set out in Tables 3–7. In Table 2, we see the summary statistics about the populations. We can also note from the numerical results presented in Tables 3–7 that the PREs of all families of estimators change with the choice of a or b. It is further noted that the proposed families of estimators are more precise than the adapted distribution function estimators of Cochran [31], Murthy [32], Rao [4], Singh et al. [33] and Grover and Kaur [11], in terms of the PRE. It can be seen that, for data sets I, III, IV and V, the second proposed family of the estimators perform better than the first proposed family of estimators, and, for data set II, it is also observed that the first proposed family of the estimators performs better than the second proposed family of estimators.

## 7 Notation in stratified random sampling

Consider a finite population Ω = {1,2,…,*N*} of *N* distinct units, which is divided into *L* homogeneous strata, where the size of *h*th stratum is *N*_*h*_, for *h* = 1,2,…,*L*, such that $\sum_{h=1}^{L}N_{h}=N$. Let *Y* and *X* be the study and auxiliary variables which take values *y*_*h*_ and *x*_*h*_, respectively, where *i* = 1,2,…,*N*_*h*_ and *h* = 1,2,…,*L*, for estimating finite population distribution function, assume a sample of size *n*_*h*_ is drawn from the hth stratum using simple random sampling without replacement, such that $\sum_{h=1}^{L}n_{h}=n$, where *n* is the sample size.

Let $F_{st}(y)=F(y)=\sum_{h=1}^{L}W_{h}F_{h}(y)$ and $F_{st}(x)=F(x)=\sum_{h=1}^{L}W_{h}F_{h}(x)$ (${\hat{F}}_{st}(y)=\hat{F}(y)=\sum_{h=1}^{L}W_{h}{\hat{F}}_{h}(y)$ and ${\hat{F}}_{st}(x)=\hat{F}(x)=\sum_{h=1}^{L}W_{h}{\hat{F}}_{h}(x)$) be the population (sample) distribution functions of *Y* and *X* under stratified random sampling, respectively, where *W*_*h*_ = *N*_*h*_/*N*, $F_{h}(y)=\sum_{i=1}^{N_{h}}I(Y_{ih}\leq y)/N_{h}$, $F_{h}(x)=\sum_{i=1}^{N_{h}}I(X_{ih}\leq y)/N_{h}$, ${\hat{F}}_{h}(y)=\sum_{i=1}^{n_{h}}I(Y_{ih}\leq y)/n_{h}$,

${\hat{F}}_{h}(x)=\sum_{i=1}^{n_{h}}I(X_{ih}\leq y)/n_{h}$. Let ${\bar{X}}_{st}=\bar{X}=\sum_{h=1}^{L}W_{h}{\bar{X}}_{h}$ and ${\bar{Z}}_{st}=\bar{Z}=\sum_{h=1}^{L}W_{h}{\bar{Z}}_{h}$ (${\hat{\bar{X}}}_{st}=\hat{\bar{X}}=\sum_{h=1}^{L}W_{h}{\hat{\bar{X}}}_{h}$ and ${\hat{\bar{Z}}}_{h}=\hat{\bar{Z}}=\sum_{i=1}^{n_{h}}Z_{ih}/n_{h}$) be the population (sample) means of *X* and *Z* under stratified random sampling, respectively, where ${\bar{X}}_{h}=\sum_{i=1}^{N_{h}}X_{ih}/N_{h}$, ${\bar{Z}}_{h}=\sum_{i=1}^{N_{h}}Z_{ih}/N_{h}$, ${\hat{\bar{X}}}_{h}=\sum_{i=1}^{n_{h}}X_{ih}/n_{h}$, ${\hat{\bar{Z}}}_{h}=\sum_{i=1}^{n_{h}}Z_{ih}/n_{h}$.

In order to obtain the biases and MSEs of the adapted and proposed estimators of *F*(*y*) under stratified random sampling, the following relative error terms are considered. Let

$$\zeta_{1}=\frac{{\hat{F}}_{st}(y)-F(y)}{F(y)},\;\zeta_{2}=\frac{{\hat{F}}_{st}(x)-F(x)}{F(x)},\;\zeta_{3}=\frac{{\hat{\bar{X}}}_{st}-\bar{X}}{\bar{X}}\mathrm{and}\;\zeta_{4}=\frac{{\hat{\bar{Z}}}_{st}-\bar{Z}}{\bar{Z}},$$

such that *E*(*e*_*i*_) = 0 for *i* = 1,2,3,4, where *E*(⋅) is the mathematical expectation of (⋅). Let

$$V_{rstu}=E[\zeta_{1}^{r}\;\zeta_{2}^{s}\;\zeta_{3}^{t}\;\zeta_{4}^{u}],$$

Where

$$E(\zeta_{1}^{2})=\sum_{h=1}^{L}W_{h}^{2}\lambda_{h}^{2}C_{1h}^{2}=\psi_{2000},E(\zeta_{2}^{2})=\sum_{h=1}^{L}W_{h}^{2}\lambda_{h}^{2}C_{2h}^{2}=\psi_{0200},$$

$$E(\zeta_{3}^{2})=\sum_{h=1}^{L}W_{h}^{2}\lambda_{h}^{2}C_{3h}^{2}=\psi_{0020},E(\zeta_{4}^{2})=\sum_{h=1}^{L}W_{h}^{2}\lambda_{h}^{2}C_{4h}^{2}=\psi_{0002},$$

$$E(\zeta_{1}\zeta_{2})=\sum_{h=1}^{L}W_{h}^{2}\lambda_{h}^{2}R_{12h}C_{1h}C_{2h}=\psi_{1100},E(\zeta_{1}\zeta_{3})=\sum_{h=1}^{L}W_{h}^{2}\lambda_{h}^{2}R_{13h}C_{1h}C_{3h}=\psi_{1010},$$

$$E(\zeta_{2}\zeta_{3})=\sum_{h=1}^{L}W_{h}^{2}\lambda_{h}^{2}R_{23h}C_{2h}C_{3h}=\psi_{0110},E(\zeta_{1}\zeta_{4})=\sum_{h=1}^{L}W_{h}^{2}\lambda_{h}^{2}R_{14h}C_{1h}C_{4h}=\psi_{1001},$$

$$E(\zeta_{2}\zeta_{4})=\sum_{h=1}^{L}W_{h}^{2}\lambda_{h}^{2}R_{24h}C_{2h}C_{4h}=\psi_{0101}.$$

## 8 Adapted estimators in stratified random sampling

In this section, some estimators of finite population mean are adapted for estimating the finite CDF under stratified random sampling. The biases and MSEs of these adapted estimators are derived under the first order of approximation.

1. The traditional unbiased estimator of *F*(*y*) is

$${\hat{F}}_{SRS_{st}}(y)=\frac{1}{n}\sum_{i=1}^{n}I(Y_{i}\leq y).$$ (32)

The variance of ${\hat{F}}_{SRS_{st}}(y)$ is

$$Var({\hat{F}}_{SRS_{st}}(y))=F^{2}(y)\psi_{2000}.$$ (33)

2. Cochran [31] adapted ratio estimator of *F*(*y*) is

$${\hat{F}}_{R_{st}}(y)={\hat{F}}_{st}(y)\left(\frac{F(x)}{{\hat{F}}_{st}(x)}\right).$$ (34)

The bias and MSE of ${\hat{F}}_{R_{st}}(y)$, to the first order of approximation, are

$$Bias({\hat{F}}_{R_{st}}(y))≅F(y)(\psi_{0200}-\psi_{1100}),$$

$$MSE({\hat{F}}_{R_{st}}(y))≅F^{2}(y)(\psi_{2000}+\psi_{0200}-2\psi_{1100}).$$ (35)

3. Murthy [32] adapted product estimator of *F*(*y*) is

$${\hat{F}}_{P_{st}}(y)={\hat{F}}_{st}(y)\left(\frac{{\hat{F}}_{st}(x)}{F(x)}\right).$$ (36)

The bias and MSE of ${\hat{F}}_{P_{st}}(y)$, to the first order of approximation, are

$$Bias({\hat{F}}_{P_{st}}(y))=F(y)\psi_{1100},$$

$$MSE({\hat{F}}_{P_{st}}(y))≅F^{2}(y)(\psi_{2000}+\psi_{0200}+2\psi_{1100}).$$ (37)

4. The adapted difference estimator of *F*(*y*) is

$${\hat{F}}_{Reg_{st}}(y)={\hat{F}}_{st}(y)+m(F(x)-{\hat{F}}_{st}(x)),$$ (38)

where *m* is an unknown constant. Here, ${\hat{F}}_{Reg_{st}}(y)$ is an unbiased estimator of *F*(*y*). The minimum variance of ${\hat{F}}_{Reg_{st}}(y)$ at the optimum value *m*_(opt)_ = (*F*(*y*)*ψ*_1100_)/(*F*(*x*)*ψ*_0200_) is

$$Var_{min}({\hat{F}}_{Reg_{st}}(y))=\frac{F^{2}(y)(\psi_{2000}\psi_{0200}-\psi_{1100}^{2})}{\psi_{0200}}.$$ (39)

Here, (39) may be written as

$$Var_{min}({\hat{F}}_{Reg_{st}}(y))=F^{2}(y)\psi_{2000}(1-R_{12}^{2}).$$ (40)

5. Rao [4] adapted difference-type estimator of *F*(*y*) is

$${\hat{F}}_{R,D_{st}}(y)=m_{1}{\hat{F}}_{st}(y)+m_{2}(F(x)-{\hat{F}}_{st}(x)),$$ (41)

where *m*_1_ and *m*_2_ are unknown constants. The bias and MSE of ${\hat{F}}_{R,D_{st}}(y)$, to the first order of approximation, are

$$Bias({\hat{F}}_{5}(y))=F(y)(m_{1}-1),$$

$$MSE({\hat{F}}_{R,D_{st}}(y))≅F^{2}(y)-2m_{1}F^{2}(y)+m_{1}^{2}F^{2}(y)+m_{1}^{2}F^{2}(y)\psi_{2000}$$

$$-2m_{1}m_{2}F(y)F(x)\psi_{1100}+m_{2}^{2}F^{2}(x)\psi_{0200}.$$ (42)

The optimum values of *m*_1_ and *m*_2_, determined by minimizing (42), are

$$m_{1(opt)}=\frac{\psi_{0200}}{\left(\psi_{0200}\psi_{2000}-\psi_{1100}^{2}+\psi_{0200}\right)},$$

$$m_{2(opt)}=\frac{F(y)\psi_{1100}}{F(x)(\psi_{2000}\psi_{0200}-\psi_{1100}^{2}+\psi_{0200})}.$$

The minimum MSE of ${\hat{F}}_{R,D_{st}}(y)$ at the optimum values of *m*_1_ and *m*_2_ is

$$MSE_{min}({\hat{F}}_{R,D_{st}}(y))=\frac{F^{2}(y)(\psi_{2000}\psi_{0200}-\psi_{1100}^{2})}{\left(\psi_{2000}\psi_{0200}-\psi_{1100}^{2}+\psi_{0200}\right)}.$$ (43)

Here, (43) may be written as

$$MSE_{min}({\hat{F}}_{R,D_{st}}(y))=\frac{F^{2}(y)\psi_{2000}(1-R_{12}^{2})}{1+\psi_{2000}(1-R_{12}^{2})}.$$ (44)

6. Singh et al. [33] adapted generalized ratio-type exponential estimator of *F*(*y*) is

$${\hat{F}}_{S_{st}}(y)={\hat{F}}_{st}(y)exp\left(\frac{a_{st}(F(x)-{\hat{F}}_{st}(x))}{a_{st}(F(x)+{\hat{F}}_{st}(x))+2b_{st}}\right),$$ (45)

where *a*_*st*_ and *b*_*st*_ are known constants. The bias and MSE of ${\hat{F}}_{S_{st}}(y)$, to the first order of approximation, are

$$Bias({\hat{F}}_{S_{st}}(y))≅F(y)(\frac{3}{8}\Theta^{2}\psi_{0200}-\frac{1}{2}\Theta \psi_{1100}),$$

$$MSE({\hat{F}}_{S_{st}}(y))≅\frac{F^{2}(y)}{4}(4\psi_{2000}+\Theta^{2}\psi_{0200}-4\Theta \psi_{1100}),$$ (46)

where *Θ* = *a*_*st*_*F*(*x*)/(*a*_*st*_*F*(*x*)+*b*_*st*_).

7. Grover and Kaur [11] adapted generalized class of ratio-type exponential estimator of *F*(*y*) is

$${\hat{F}}_{G,K_{st}}(y)=\{m_{3}{\hat{F}}_{st}(y)+m_{4}(F(x)-{\hat{F}}_{st}(x))\}exp\left(\frac{a_{st}(F(x)-{\hat{F}}_{st}(x))}{a_{st}(F(x)+{\hat{F}}_{st}(x))+2b_{st}}\right),$$ (47)

where *m*_3_ and *m*_4_ are unknown constants. The bias and MSE of ${\hat{F}}_{G,K_{st}}(y)$, to the first order of approximation, are

$$Bias({\hat{F}}_{G,K_{st}}(y))≅F(y)(m_{3}-1)+\frac{3}{8}\Theta^{2}m_{3}F(y)+\frac{1}{2}\Theta m_{4}F(x)\psi_{0200}-\frac{1}{2}\Theta F(y)\psi_{1100},$$

$$MSE({\hat{F}}_{G,K_{st}}(y))≅m_{4}^{2}F^{2}(x)\psi_{0200}+m_{3}^{2}F^{2}(y)\psi_{2000}+2\Theta m_{3}m_{4}F(y)F(x)\psi_{0200}$$

$$-2m_{3}m_{4}F(y)F(x)\psi_{1100}+F^{2}(y)-2m_{3}F^{2}(y)+\Theta m_{3}^{2}F^{2}(y)$$

$$+m_{3}F^{2}(y)\psi_{1100}-\Theta m_{4}F(y)F(x)\psi_{0200}-2\Theta m_{3}^{2}F^{2}(y)\psi_{1100}$$

$$-\frac{3}{4}\Theta^{2}m_{3}F^{2}(y)\psi_{0200}+\Theta^{2}m_{3}^{2}F^{2}(y)\psi_{0200}.$$ (48)

The optimum values of *m*_3_ and *m*_4_, determined by minimizing (48), are

$$m_{3(opt)}=\frac{\psi_{0200}(\Theta^{2}\psi_{0200}-8)}{8(-\psi_{2000}\psi_{0200}+\psi_{1100}^{2}-\psi_{0200})},$$

$$m_{4(opt)}=\frac{F(y)(\Theta^{3}\psi_{0200}^{2}-\Theta^{2}\psi_{0200}\psi_{1100}+4\Theta \psi_{2000}\psi_{0200}-4\Theta \psi_{1100}^{2}-4\Theta \psi_{0200}+8\psi_{1100})}{8F(x)(\psi_{2000}\psi_{0200}-\psi_{1100}^{2}+\psi_{0200})}.$$

The simplified minimum MSE of ${\hat{F}}_{G,K_{st}}(y)$ at the optimum values of *m*_3_ and *m*_4_ is

$$MSE_{min}({\hat{F}}_{G,K_{st}}(y))≅\frac{F^{2}(y)}{64}\left(64-16\Theta^{2}\psi_{0200}-\frac{\psi_{0200}(-8+\Theta^{2}\psi_{0200}{)}^{2}}{\psi_{0200}(1+\psi_{2000})-V_{1100}^{2}}\right).$$ (49)

Here, (49) may be written as

$$MSE_{min}({\hat{F}}_{G,K_{st}}(y))≅Var_{min}({\hat{F}}_{Reg_{st}}(y))-\frac{F^{2}(y){\left(\Theta^{2}\psi_{0200}^{2}-8\psi_{1100}^{2}+8\psi_{0200}\psi_{2000}\right)}^{2}}{64\psi_{0200}^{2}\{1+\psi_{2000}(1-R_{12}^{2})\}},$$ (50)

which shows that ${\hat{F}}_{G,K_{st}}(y)$ is more precise than ${\hat{F}}_{Reg_{st}}(y)$.

## 9 Proposed estimators in stratified random sampling

### 9.1 First proposed family of estimators

On the lines of ${\hat{F}}_{R,D_{st}}(y)$ and ${\hat{F}}_{S_{st}}(y)$, first proposed family of estimators for estimating *F*(*y*) is given by

$${\hat{F}}_{Prop1_{st}}(y)=\left\{m_{5}{\hat{F}}_{st}(y)+m_{6}(\frac{F(x)-{\hat{F}}_{st}(x)}{F(x)})+m_{7}(\frac{\bar{X}-{\hat{\bar{X}}}_{st}}{\bar{X}})\right\}exp\left(\frac{a_{st}(F(x)-{\hat{F}}_{st}(x))}{a_{st}(F(x)+{\hat{F}}_{st}(x))+2b_{st}}\right),$$ (51)

where *m*_5_, *m*_6_ and *m*_7_ are unknown constants, *a*_*st*_(≠0) and *b*_*st*_ are either two real numbers or functions of known population parameters of *I*(*X*≤*x*), like *R*_12_, $\beta_{2(st)}=\sum_{i=1}^{L}W_{h}\beta_{2h(x)}$ (coefficient of kurtosis), $C_{2}=\sum_{i=1}^{L}W_{h}C_{2h}$, etc.

The estimator ${\hat{F}}_{Prop1_{st}}(y)$ can also be written as

$${\hat{F}}_{Prop1_{st}}(y)=\{m_{5}F(y)(1+\zeta_{1})-m_{6}\zeta_{2}-m_{7}\zeta_{3}\}(1-\frac{1}{2}\Theta \zeta_{2}+\frac{3}{8}\Theta^{2}\zeta_{2}^{2}+\cdots ).$$ (52)

Simplifying (52) and keeping terms only up to the second power of *ζ*_*i*_’s, we can write

$$({\hat{F}}_{Prop1_{st}}(y)-F(y))=-F(y)+m_{5}F(y)+m_{5}F(y)\zeta_{1}-\frac{1}{2}\Theta m_{5}F(y)\zeta_{2}-m_{6}\zeta_{2}-m_{7}\zeta_{3}$$

$$+\frac{3}{8}\Theta^{2}m_{5}F(y)\zeta_{2}^{2}+\frac{1}{2}\Theta m_{6}\zeta_{2}^{2}-\frac{1}{2}\Theta m_{5}F(y)\zeta_{1}\zeta_{2}+\frac{1}{2}\Theta m_{7}\zeta_{2}\zeta_{3}.$$ (53)

The bias and MSE of ${\hat{F}}_{Prop1_{st}}(y)$, to the first order of approximation, are

$$Bias({\hat{F}}_{Prop1_{st}}(y))≅F(y)(m_{5}-1)+\frac{3}{8}\Theta^{2}m_{5}F(y)\psi_{0200}+\frac{1}{2}\Theta m_{6}\psi_{0200}-\frac{1}{2}\Theta m_{5}F(y)\psi_{1100}+\frac{1}{2}\Theta m_{7}\psi_{0110},$$

$$MSE({\hat{F}}_{Prop1_{st}}(y))≅F^{2}(y)(m_{5}-1{)}^{2}+m_{5}^{2}F^{2}(y)\psi_{2000}+m_{6}^{2}\psi_{0200}+m_{7}^{2}\psi_{0020}+\Theta^{2}k_{5}^{2}F^{2}(y)\psi_{0200}$$

$$-\Theta m_{6}F(y)\psi_{0200}+2\Theta m_{5}m_{6}F(y)\psi_{0200}-\frac{3}{4}\Theta^{2}m_{5}F^{2}(y)\psi_{0200}+\Theta m_{5}F^{2}(y)\psi_{1100}$$

$$-2\Theta k_{5}^{2}F^{2}(y)\psi_{1100}-2m_{5}m_{6}F(y)\psi_{1100}-2m_{5}m_{7}F(y)\psi_{1010}-\Theta m_{7}F(y)\psi_{0110}$$

$$+2\Theta m_{5}m_{7}F(y)\psi_{0110}-2m_{6}m_{7}\psi_{0110}.$$ (54)

The optimum values of *m*_5_, *m*_6_ and *m*_7_, determined by minimizing (54), are

$$m_{5(opt)}=\frac{8-\Theta^{2}\psi_{0200}}{8\{1+\psi_{2000}(1-R_{1.23}^{2})\}},$$

$$m_{6(opt)}=\frac{F(y)[\begin{array}{ll} & \Theta^{3}\psi_{0200}^{3/2}(R_{23}^{2}-1)+\psi_{2000}^{1/2}(-8+\Theta^{2}\psi_{0200})(R_{12}-R_{23}R_{13})\\ & +4\Theta \psi_{0200}^{1/2}(R_{23}^{2}-1)\{-1+\psi_{2000}(1-R_{1.23}^{2})\} \end{array}]}{8\psi_{0200}^{1/2}(R_{23}^{2}-1)\{-1+\psi_{2000}(1-R_{1.23}^{2})\}},$$

$$m_{7(opt)}=\frac{F(y)\psi_{2000}^{1/2}(8-\Theta^{2}\psi_{0200})(R_{12}-R_{23}R_{13})}{8\psi_{0200}^{1/2}(R_{23}^{2}-1)\{-1+\psi_{2000}(1-R_{1.23}^{2})\}}.$$

The simplified minimum MSE of ${\hat{F}}_{Prop1_{st}}(y)$ at the optimum values of *m*_5_, *m*_6_ and *m*_7_ is

$$MSE_{min}({\hat{F}}_{Prop1_{st}}(y))≅\frac{F^{2}(y)\{64\psi_{2000}(1-R_{1.23}^{2})-\Theta^{4}\psi_{0200}^{2}-16\Theta^{2}\psi_{0200}\psi_{2000}(1-R_{1.23}^{2})\}}{64\{1+\psi_{2000}(1-R_{1.23}^{2})\}},$$ (55)

where $R_{1.23}^{2}=\left(\frac{\psi_{1100}^{2}\psi_{0020}+\psi_{1010}^{2}\psi_{0200}-2\psi_{1010}\psi_{1100}\psi_{0110}}{\psi_{2000}(\psi_{0200}\psi_{0020}-\psi_{0110}^{2})}\right)$.

Here, (55) may be written as

$$MSE_{min}({\hat{F}}_{Prop1_{st}}(y))≅Var_{min}({\hat{F}}_{Reg_{st}}(y))-T_{1}-T_{2}$$ (56)

where

$$T_{1}=\frac{F^{2}(y){\left(\Theta^{2}\psi_{0200}^{2}-8\psi_{1100}^{2}+8\psi_{0200}\psi_{2000}\right)}^{2}}{64\psi_{0200}^{2}\{1+\psi_{2000}(1-R_{12}^{2})\}}and$$

$$T_{2}=\frac{F^{2}(y){\left(\Theta^{2}\psi_{0200}-8\right)}^{2}{\left(\psi_{0200}\psi_{1010}-\psi_{0110}\psi_{1100}\right)}^{2}}{64\psi_{0200}^{2}\psi_{0020}(1-R_{23}^{2})\{1+\psi_{2000}(1-R_{12}^{2})\}\{1+\psi_{2000}(1-R_{1.23}^{2})\}}.$$

It can be seen that ${\hat{F}}_{Prop1_{st}}(y)$ is more precise than ${\hat{F}}_{Reg_{st}}(y).$

### 9.2 Second proposed family of estimators

On similar lines, second proposed family of estimators for estimating *F*(*y*) is given by

$${\hat{F}}_{Prop2_{st}}(y)=\left\{m_{8}{\hat{F}}_{st}(y)+m_{9}(\frac{F(x)-{\hat{F}}_{st}(x)}{F(x)})+m_{10}(\frac{\bar{Z}-{\hat{\bar{Z}}}_{st}}{\bar{Z}})\right\}exp(\frac{a_{st}(F(x)-{\hat{F}}_{st}(x))}{a_{st}(F(x)-{\hat{F}}_{st}(x))+2b_{st}}),$$ (57)

where *m*_8_, *m*_9_ and *m*_10_ are unknown constants, *a*_*st*_ (≠0) and *b*_*st*_ are either two real numbers or functions of known population parameters of *I*(*X*≤*x*), like *R*_12_, $\beta_{2(st)}=\sum_{i=1}^{L}W_{h}\beta_{2h(x)}$ (coefficient of kurtosis), $C_{2}=\sum_{i=1}^{L}W_{h}C_{2h}$, etc.

The estimator ${\hat{F}}_{Prop2_{st}}(y)$ can also be written as

$${\hat{F}}_{Prop2_{st}}(y)=\{m_{8}F(y)(1+\zeta_{1})-m_{9}\zeta_{2}-m_{10}\zeta_{4}\}(1-\frac{1}{2}\Theta \zeta_{2}+\frac{3}{8}\Theta^{2}\zeta_{2}^{2}+\cdots ).$$ (58)

Simplifying (58) and keeping terms only up to the second power of *ζ*_*i*_’s, we can write

$$({\hat{F}}_{Prop2_{st}}(y)-F(y))=-F(y)+m_{8}F(y)+m_{8}F(y)\zeta_{1}-\frac{1}{2}\Theta m_{8}F(y)\zeta_{2}-m_{9}\zeta_{2}-m_{10}\zeta_{4}$$

$$+\frac{3}{8}\Theta^{2}m_{8}F(y)\zeta_{2}^{2}+\frac{1}{2}\Theta m_{9}\zeta_{2}^{2}-\frac{1}{2}\Theta m_{8}F(y)\zeta_{1}\zeta_{2}+\frac{1}{2}\Theta m_{10}\zeta_{2}\zeta_{4}.$$ (59)

The bias and MSE of ${\hat{F}}_{Prop2_{st}}(y)$, to the first order of approximation, are

$$Bias({\hat{F}}_{Prop2_{st}}(y))≅F(y)(m_{8}-1)+\frac{3}{8}\Theta^{2}m_{8}F(y)\psi_{0200}+\frac{1}{2}\Theta m_{9}\psi_{0200}-\frac{1}{2}\Theta m_{8}F(y)\psi_{1100}+\frac{1}{2}\Theta m_{9}\psi_{0101},$$

$$MSE({\hat{F}}_{Prop2_{st}}(y))≅F^{2}(y)(m_{8}-1{)}^{2}+m_{8}^{2}F^{2}(y)\psi_{2000}+m_{9}^{2}\psi_{0200}+m_{10}^{2}\psi_{0002}+\Theta^{2}k_{8}^{2}F^{2}(y)\psi_{0200}$$

$$-\Theta m_{9}F(y)\psi_{0200}+2\Theta m_{8}m_{9}F(y)\psi_{0200}-\frac{3}{4}\Theta^{2}m_{8}F^{2}(y)\psi_{0200}+\Theta m_{8}F^{2}(y)\psi_{1100}$$

$$-2\Theta k_{8}^{2}F^{2}(y)\psi_{1100}-2m_{8}m_{9}F(y)\psi_{1100}-2m_{8}m_{10}F(y)\psi_{1001}-\Theta m_{10}F(y)\psi_{0101}$$

$$+2\Theta m_{8}m_{10}F(y)\psi_{0101}-2m_{9}m_{10}\psi_{0101}.$$ (60)

The optimum values of *m*_8_, *m*_9_ and *m*_10_, determined by minimizing (60), are

$$m_{8(opt)}=\frac{8-\Theta^{2}\psi_{0200}}{8\{1+\psi_{2000}(1-R_{1.24}^{2})\}},$$

$$m_{9(opt)}=\frac{F(y)[\begin{array}{ll} & \Theta^{3}\psi_{0200}^{3/2}(R_{24}^{2}-1)+\psi_{2000}^{1/2}(-8+\Theta^{2}\psi_{0200})(R_{12}-R_{24}R_{14})\\ & +4\Theta \psi_{0200}^{1/2}(R_{24}^{2}-1)\{-1+\psi_{2000}(1-R_{1.24}^{2})\} \end{array}]}{8\psi_{0200}^{1/2}(R_{24}^{2}-1)\{-1+\psi_{2000}(1-R_{1.24}^{2})\}},$$

$$m_{10(opt)}=\frac{F(y)\psi_{2000}^{1/2}(8-\Theta^{2}\psi_{0200})(R_{12}-R_{24}R_{14})}{8\psi_{0200}^{1/2}(R_{24}^{2}-1)\{-1+\psi_{2000}(1-R_{1.24}^{2})\}}.$$

The simplified minimum MSE of ${\hat{F}}_{Prop2_{st}}(y)$ at the optimum values of *m*_8_, *m*_9_ and *m*_10_ is

$$MSE_{min}({\hat{F}}_{Prop2_{st}}(y))≅\frac{F^{2}(y)\{64\psi_{2000}(1-R_{1.24}^{2})-\Theta^{4}\psi_{0200}^{2}-16\Theta \psi_{0200}\psi_{2000}(1-R_{1.24}^{2})\}}{64\{1+\psi_{2000}(1-R_{1.24}^{2})\}},$$ (61)

where $R_{1.24}^{2}=\left(\frac{\psi_{1100}^{2}\psi_{0002}+\psi_{1001}^{2}\psi_{0200}-2\psi_{1001}\psi_{1100}\psi_{0101}}{\psi_{2000}(\psi_{0200}\psi_{0002}-\psi_{0101}^{2})}\right)$.

Here, (61) may be written as

$$MSE_{min}({\hat{F}}_{Prop2_{st}}(y))≅Var_{min}({\hat{F}}_{Reg_{st}}(y))-T_{1}-T_{3},$$ (62)

where

$$T_{1}=\frac{F^{2}(y){\left(\Theta^{2}\psi_{0200}^{2}-8\psi_{1100}^{2}+8\psi_{0200}\right)}^{2}}{64\psi_{0200}^{2}\{1+\psi_{2000}(1-R_{12}^{2})\}}and$$

$$T_{3}=\frac{F^{2}(y){\left(\Theta^{2}\psi_{0200}-8\right)}^{2}{\left(\psi_{0200}\psi_{1001}-\psi_{0101}\psi_{1100}\right)}^{2}}{64\psi_{0200}^{2}\psi_{0002}(1-R_{24}^{2})\{1+\psi_{2000}(1-R_{12}^{2})\}\{1+\psi_{2000}(1-R_{1.24}^{2})\}}.$$

respectively, which shows that ${\hat{F}}_{Prop2_{st}}(y)$ is more precise than ${\hat{F}}_{Reg_{st}}(y)$.

In Table 8, we put some members of the Singh et al. [33], Grover and Kaur [11], and proposed families of estimators with selected choices of *a* and *b*.

**Table 8:** Some members of the proposed and adapted estimators.

*a*	*b*	${\hat{F}}_{S_{st}}(y)$	${\hat{F}}_{G,K_{st}}(y)$	${\hat{F}}_{Prop1_{st}}(y)$	${\hat{F}}_{9}(y)$
1	*C*_2_	${\hat{F}}_{S_{st}}^{\left(1\right)}(y)$	${\hat{F}}_{G,K_{st}}^{\left(1\right)}(y)$	${\hat{F}}_{Prop1_{st}}^{\left(1\right)}(y)$	${\hat{F}}_{Prop2_{st}}^{\left(1\right)}(y)$
1	*β*_2(*st*)_	${\hat{F}}_{S_{st}}^{\left(2\right)}(y)$	${\hat{F}}_{G,K_{st}}^{\left(2\right)}(y)$	${\hat{F}}_{Prop1_{st}}^{\left(2\right)}(y)$	${\hat{F}}_{Prop2_{st}}^{\left(2\right)}(y)$
*β*_2(*st*)_	*C*_2_	${\hat{F}}_{S_{st}}^{\left(3\right)}(y)$	${\hat{F}}_{G,K_{st}}^{\left(3\right)}(y)$	${\hat{F}}_{Prop1_{st}}^{\left(3\right)}(ty)$	${\hat{F}}_{Prop2_{st}}^{\left(3\right)}(y)$
$C_{2}$	*β*_2(*st*)_	${\hat{F}}_{S_{st}}^{\left(4\right)}(y)$	${\hat{F}}_{G,K_{st}}^{\left(4\right)}(y)$	${\hat{F}}_{Prop1_{st}}^{\left(4\right)}(y)$	${\hat{F}}_{Prop2_{st}}^{\left(4\right)}(y)$
1	*R*_12_	${\hat{F}}_{S_{st}}^{\left(5\right)}(y)$	${\hat{F}}_{G,K_{st}}^{\left(5\right)}(y)$	${\hat{F}}_{Prop1_{st}}^{\left(5\right)}(y)$	${\hat{F}}_{Prop2_{st}}^{\left(5\right)}(y)$
*C*_2_	*R*_12_	${\hat{F}}_{S_{st}}^{\left(6\right)}(y)$	${\hat{F}}_{G,K_{st}}^{\left(6\right)}(y)$	${\hat{F}}_{Prop1_{st}}^{\left(6\right)}(y)$	${\hat{F}}_{Prop2_{st}}^{\left(6\right)}(y)$
*R*_12_	*C*_2_	${\hat{F}}_{S_{st}}^{\left(7\right)}(y)$	${\hat{F}}_{G,K_{st}}^{\left(7\right)}(y)$	${\hat{F}}_{Prop1_{st}}^{\left(7\right)}(y)$	${\hat{F}}_{Prop2_{st}}^{\left(7\right)}(y)$
*β*_2(*st*)_	*R*_12_	${\hat{F}}_{S_{st}}^{\left(8\right)}(y)$	${\hat{F}}_{G,K_{st}}^{\left(8\right)}(y)$	${\hat{F}}_{Prop1_{st}}^{\left(8\right)}(y)$	${\hat{F}}_{Prop2_{st}}^{\left(8\right)}(y)$
*R*_12_	*β*_2(*st*)_	${\hat{F}}_{S_{st}}^{\left(9\right)}(y)$	${\hat{F}}_{G,K_{st}}^{\left(9\right)}(y)$	${\hat{F}}_{Prop1_{st}}^{\left(9\right)}(y)$	${\hat{F}}_{Prop2_{st}}^{\left(9\right)}(y)$
1	*NF*(*x*)	${\hat{F}}_{S_{st}}^{\left(10\right)}(y)$	${\hat{F}}_{G,K_{st}}^{\left(10\right)}(y)$	${\hat{F}}_{Prop1_{st}}^{\left(10\right)}(y)$	${\hat{F}}_{Prop2_{st}}^{\left(10\right)}(y)$

## 10 Efficiency comparisons in stratified random sampling

In this section, the adapted and proposed estimators of *F*(*y*) are compared in terms of the minimum MSEs. [(i)]

1. From (33) and (56),

$$MSE_{min}({\hat{F}}_{Prop1_{st}}(y))<Var({\hat{F}}_{SRS_{st}}(y))if$$

$$F^{2}(y)\psi_{2000}R_{12}^{2}+T_{1}+T_{2}>0.$$

2. From (35) and (56),

$$MSE_{min}({\hat{F}}_{Prop1_{st}}(y))<MSE({\hat{F}}_{R_{st}}(y))if$$

$$\frac{F^{2}(y)}{\psi_{2000}}{\left(\psi_{0200}-\psi_{1100}\right)}^{2}+T_{1}+T_{2}>0.$$

3. From (37) and (56),

$$MSE_{min}({\hat{F}}_{Prop1_{st}}(y))<MSE({\hat{F}}_{P_{st}}(y))if$$

$$\frac{F^{2}(y)}{\psi_{2000}}{\left(\psi_{0200}+\psi_{1100}\right)}^{2}+T_{1}+T_{2}>0.$$

4. From (40) and (56),

$$MSE_{min}({\hat{F}}_{Prop1_{st}}(y))<MSE_{min}({\hat{F}}_{Reg_{st}}(y))if$$

$$T_{1}+T_{2}>0.$$

5. From (44) and (56),

$$MSE_{min}({\hat{F}}_{Prop1_{st}}(y))<MSE_{min}({\hat{F}}_{R,D_{st}}(y))if$$

$$\frac{F^{2}(y)\Theta^{2}\psi_{0200}\{\Theta^{2}\psi_{0200}+16\psi_{2000}(1-R_{12}^{2})\}}{64\{1+\psi_{2000}(1-R_{12}^{2})\}}+T_{2}>0.$$

6. From (46) and (56),

$$MSE_{min}({\hat{F}}_{Prop1_{st}}(y))<MSE({\hat{F}}_{S_{st}}(y))if$$

$$\frac{F^{2}(y)}{\psi_{2000}}{\left(\frac{\Theta \psi_{0200}}{2}-\psi_{1100}\right)}^{2}+T_{1}+T_{2}>0.$$

7. From (50) and (56),

$$MSE_{min}({\hat{F}}_{Prop1_{st}}(y))<MSE_{min}({\hat{F}}_{G,K_{st}}(y))if$$

$$T_{2}>0.$$

8. From (33) and (62),

$$MSE_{min}({\hat{F}}_{Prop2_{st}}(y))<Var({\hat{F}}_{SRS_{st}}(y))if$$

$$\frac{1}{\psi_{0200}}F^{2}(y)\psi_{1100}^{2}+T_{1}+T_{3}>0.$$

9. From (35) and (62),

$$MSE_{min}({\hat{F}}_{Prop2_{st}}(y))<MSE({\hat{F}}_{R_{st}}(y))if$$

$$\frac{F^{2}(y)}{\psi_{2000}}{\left(\psi_{0200}-\psi_{1100}\right)}^{2}+T_{1}+T_{3}>0.$$

10. From (37) and (62),

$$MSE_{min}({\hat{F}}_{Prop2_{st}}(y))<MSE({\hat{F}}_{P_{st}}(y))if$$

$$\frac{F^{2}(y)}{\psi_{2000}}{\left(\psi_{0200}+\psi_{1100}\right)}^{2}+T_{1}+T_{3}>0.$$

11. From (40) and (62),

$$MSE_{min}({\hat{F}}_{Prop2_{st}}(y))<MSE_{min}({\hat{F}}_{Reg_{st}}(y))if$$

$$T_{1}+T_{3}>0.$$

12. From (44) and (62),

$$MSE_{min}({\hat{F}}_{Prop2_{st}}(y))<MSE_{min}({\hat{F}}_{R,D_{st}}(y))if$$

$$\frac{F^{2}(y)\Theta^{2}\psi_{0200}\{\Theta^{2}\psi_{0200}+16\psi_{2000}(1-R_{12}^{2})\}}{64\{1+\psi_{2000}(1-R_{12}^{2})\}}+T_{3}>0.$$

13. From (46) and (62),

$$MSE_{min}({\hat{F}}_{Prop2_{st}}(y))<MSE_{min}({\hat{F}}_{S_{st}}(y))if$$

$$\frac{F^{2}(y)}{\psi_{2000}}{\left(\frac{\Theta \psi_{0200}}{2}-\psi_{1100}\right)}^{2}+T_{1}+T_{3}>0.$$

14. From (50) and (62),

$$MSE_{min}({\hat{F}}_{Prop2_{st}}(y))<MSE_{min}({\hat{F}}_{G,K_{st}}(y))if$$

$$T_{3}>0.$$

The proposed families of estimators are always more precise than the adapted estimators as the above conditions (i)–(xiv) are always true.

## 11 Empirical study in stratified random sampling

In this section, we conduct a numerical study to investigate the performances of the adapted and proposed CDF estimators. For this purpose, five populations are considered. The summary statistics of these populations are reported in Tables 9–12. The PRE of an estimator ${\hat{F}}_{i}(y)$ with respect to ${\hat{F}}_{1}(y)$ is

$$PRE({\hat{F}}_{i}(y),{\hat{F}}_{1}(y))=\frac{Var({\hat{F}}_{1}(y))}{MSE_{min}({\hat{F}}_{i}(y))}\times 100$$

where *i* = 2,3,…,9.

**Table 9:** Summary statistics for Population I.

*h*	*N*_*h*_	*n*_*h*_	*W*_*h*_	*λ*_*h*_	*F*(*y*_*h*_)	*F*(*x*_*h*_)	${\bar{X}}_{h}$	${\bar{Z}}_{h}$
1	127	31	0.1375	0.0244	0.3543	0.3779	20805	64
2	117	21	0.1267	0.0390	0.4188	0.4872	9212	59
3	103	29	0.1115	0.0248	0.4272	0.4660	14309	52
4	170	38	0.1841	0.0204	0.5765	0.6118	9479	86
5	205	22	0.2221	0.0406	0.6146	0.6537	5570	103
6	201	39	0.2177	0.0207	0.5025	0.3532	12998	101
*S*_1*h*_	*S*_2*h*_	*S*_3*h*_	*S*_4*h*_	*R*_12*h*_	*R*_13*h*_	*R*_23*h*_	*R*_14*h*_	*R*_24*h*_
.4802	0.4868	30487	36.806	0.9164	−0.4602	−0.4832	−0.8155	−0.8399
.4955	0.5019	15181	33.919	0.8709	−0.4147	−0.4600	−0.8437	−0.8658
.4970	0.5013	27550	29.877	0.9244	−0.3928	−0.4198	−0.8489	−0.8640
.4956	0.4888	18219	49.219	0.8805	−0.5074	−0.5396	−0.8417	−0.8441
.4879	0.4769	8498	59.322	0.8772	−0.5579	−0.5909	−0.8305	−0.8241
.5012	0.4792	23094	58.168	0.7145	−0.4334	−0.3554	−0.8125	−0.8279

**Table 10:** Summary statistics for Population II.

*h*	*N*_*h*_	*n*_*h*_	*W*_*h*_	*λ*_*h*_	*F*(*y*_*h*_)	*F*(*x*_*h*_)	${\bar{X}}_{h}$	${\bar{Z}}_{h}$
1	127	31	0.1375	0.0244	0.3543	0.3700	498.276	64
2	117	21	0.1267	0.0391	0.4188	0.4700	318.333	59
3	103	29	0.1115	0.0248	0.4272	0.4272	431.359	52
4	170	38	0.1841	0.0204	0.5765	0.5882	311.324	86
5	205	22	0.2221	0.0406	0.6146	0.6146	227.195	103
6	201	39	0.2177	0.0207	0.5025	0.4527	312.706	101
*S*_1*h*_	*S*_2*h*_	*S*_3*h*_	*S*_4*h*_	*R*_12*h*_	*R*_13*h*_	*R*_23*h*_	*R*_14*h*_	*R*_24*h*_
.4802	0.4847	555.58	36.805	0.8983	−0.5398	−0.5580	−0.8114	−0.8363
.4955	0.5013	365.45	33.918	0.8666	−0.5205	−0.5589	−0.8382	−0.8645
.4970	0.4970	613.95	29.877	0.9603	−0.4803	−0.4823	−0.8519	−0.8568
.4956	0.4936	458.02	49.217	0.9277	−0.5818	−0.5934	−0.8490	−0.8525
.4879	0.4879	260.85	59.321	0.8764	−0.6395	−0.6457	−0.8285	−0.8429
.5012	0.4990	397.04	58.167	0.8450	−0.5063	−0.4873	−0.8343	−0.8622

**Table 11:** Summary statistics for Population III.

*h*	*N*_*h*_	*n*_*h*_	*W*_*h*_	*λ*_*h*_	*F*(*y*_*h*_)	*F*(*x*_*h*_)	${\bar{X}}_{h}$	${\bar{Z}}_{h}$
1	106	9	0.1241	0.1017	0.5849	0.5472	24376	54
2	106	17	0.1241	0.0494	0.5189	0.5660	27422	54
3	94	38	0.1100	0.0157	0.3298	0.3404	72410	48
4	171	67	0.2002	0.0090	0.3684	0.3801	74365	87
5	204	7	0.2389	0.1379	0.4657	0.4657	26442	103
6	173	2	0.2026	0.4942	0.7052	0.7225	9844	87
*S*_1*h*_	*S*_2*h*_	*S*_3*h*_	*S*_4*h*_	*R*_12*h*_	*R*_13*h*_	*R*_23*h*_	*R*_14*h*_	*R*_24*h*_
.4950	0.5001	49189	30.743	0.7722	−0.4470	−0.4523	−0.7665	−0.8622
.5020	0.4979	5746	30.743	0.8330	−0.4370	−0.4816	−0.8285	−0.8585
.4727	0.4764	160757	27.279	0.7854	−0.2957	−0.3087	−0.7509	−0.8208
.4838	0.4868	285603	49.507	0.7755	−0.1848	−0.1936	−0.7535	−0.8408
.5000	0.4965	45403	59.033	0.6750	−0.3929	−0.4129	−0.7218	−0.8578
.4573	0.4490	18794	50.084	0.7319	−0.5598	−0.6102	−0.7290	−0.7755

**Table 12:** Summary statistics for Population IV.

*h*	*N*_*h*_	*n*_*h*_	*W*_*h*_	*λ*_*h*_	*F*(*y*_*h*_)	*F*(*x*_*h*_)	${\bar{X}}_{h}$	${\bar{Z}}_{h}$
1	106	9	0.1241	0.1017	0.5849	0.5189	24712	54
2	106	17	0.1241	0.0494	0.5189	0.5660	26840	54
3	94	38	0.1100	0.0157	0.3298	0.3404	72722	48
4	171	67	0.2002	0.0090	0.3684	0.3743	73191	87
5	204	7	0.2389	0.1379	0.4657	0.4363	26834	103
6	173	2	0.2026	0.4942	0.7052	0.7341	9903	87
*S*_1*h*_	*S*_2*h*_	*S*_3*h*_	*S*_4*h*_	*R*_12*h*_	*R*_13*h*_	*R*_23*h*_	*R*_14*h*_	*R*_24*h*_
.4950	0.5020	49135	30.743	0.7598	−0.4439	−0.4360	−0.7474	−0.8655
.5020	0.4979	53979	30.743	0.8330	−0.5011	−0.4816	−0.8303	−0.8585
.4727	0.4764	161110	27.279	0.7376	−0.2957	−0.3093	−0.7460	−0.8208
.4838	0.4854	26249	49.507	0.7871	−0.1974	−0.2050	−0.7516	−0.8382
.5000	0.4971	45174	59.033	0.6690	−0.4011	−0.4266	−0.7164	−0.8590
.4573	0.4430	18977	50.084	0.7299	−0.5399	−0.6241	−0.7002	−0.7653

The PREs of distribution function estimators, computed from four populations, are given in Tables 9–12.

**Population I** (Source: Koyuncu and Kadilar, [36])

*Y*: The number of teachers and

*X*: The number of students in both primary and secondary schools in Turkey in 2007 for 923 districts in six regions.

**Population II** (Source: Koyuncu and Kadilar, [36])

*Y*: The number of teachers and

*X*: The number of classes in both primary and secondary schools in Turkey in 2007 for 923 districts in six regions.

**Population III** (Source: Kadilar and Cingi, [37])

*Y*: Apple production amount in 1999 and

*X*: The number of apple trees in 1999.

**Population IV** (Source: Kadilar and Cingi, [37])

*Y*: Apple production amount in 1999 and

*X*: Apple production amount in 1998.

From the numerical results, presented in Tables 13–16, it is observed that the PREs of all families of estimators change with the choices of *a* and *b*. It is further noted that the proposed families of estimators are more precise than the adapted distribution function estimators of Cochran [31], Murthy [32], Rao [4], Singh et al. [33] and Grover and Kaur [11], in terms of PRE. It can be seen that, for all data sets, the second proposed family of the estimators perform better than the first proposed family of estimators.

**Table 13:** PREs of distribution function estimators using Population I.

Estimator	Value	Estimator	Value	Estimator	Value	Estimator	Value	Estimator	Value
${\hat{F}}_{1}(y)$	100	${\hat{F}}_{6}^{\left(1\right)}(y)$	133.03	${\hat{F}}_{7}^{\left(1\right)}(y)$	364.28	${\hat{F}}_{8}^{\left(1\right)}(y)$	370.30	${\hat{F}}_{9}^{\left(1\right)}(y)$	428.83
${\hat{F}}_{2}(y)$	341.19	${\hat{F}}_{6}^{\left(2\right)}(y)$	128.23	${\hat{F}}_{7}^{\left(2\right)}(y)$	364.27	${\hat{F}}_{8}^{\left(2\right)}(y)$	370.29	${\hat{F}}_{9}^{\left(2\right)}(y)$	428.82
${\hat{F}}_{3}(y)$	27.38	${\hat{F}}_{6}^{\left(3\right)}(y)$	139.35	${\hat{F}}_{7}^{\left(3\right)}(y)$	364.29	${\hat{F}}_{8}^{\left(3\right)}(y)$	370.32	${\hat{F}}_{9}^{\left(3\right)}(y)$	428.85
${\hat{F}}_{4}(y)$	363.74	${\hat{F}}_{6}^{\left(4\right)}(y)$	128.99	${\hat{F}}_{7}^{\left(4\right)}(y)$	364.27	${\hat{F}}_{8}^{\left(4\right)}(y)$	370.30	${\hat{F}}_{9}^{\left(4\right)}(y)$	428.82
${\hat{F}}_{5}(y)$	364.23	${\hat{F}}_{6}^{\left(5\right)}(y)$	138.40	${\hat{F}}_{7}^{\left(5\right)}(y)$	364.29	${\hat{F}}_{8}^{\left(5\right)}(y)$	370.32	${\hat{F}}_{9}^{\left(5\right)}(y)$	428.85
		${\hat{F}}_{6}^{\left(6\right)}(y)$	139.35	${\hat{F}}_{7}^{\left(6\right)}(y)$	364.29	${\hat{F}}_{8}^{\left(6\right)}(y)$	370.32	${\hat{F}}_{9}^{\left(6\right)}(y)$	428.85
		${\hat{F}}_{6}^{\left(7\right)}(y)$	128.99	${\hat{F}}_{7}^{\left(7\right)}(y)$	364.27	${\hat{F}}_{8}^{\left(7\right)}(y)$	370.29	${\hat{F}}_{9}^{\left(7\right)}(y)$	428.82
		${\hat{F}}_{6}^{\left(8\right)}(y)$	145.36	${\hat{F}}_{7}^{\left(8\right)}(y)$	364.31	${\hat{F}}_{8}^{\left(8\right)}(y)$	370.34	${\hat{F}}_{9}^{\left(8\right)}(y)$	428.87
		${\hat{F}}_{6}^{\left(9\right)}(y)$	124.67	${\hat{F}}_{7}^{\left(9\right)}(y)$	364.26	${\hat{F}}_{8}^{\left(9\right)}(y)$	370.29	${\hat{F}}_{9}^{\left(9\right)}(y)$	428.81
		${\hat{F}}_{6}^{\left(10\right)}(y)$	100.09	${\hat{F}}_{7}^{\left(10\right)}(y)$	364.23	${\hat{F}}_{8}^{\left(10\right)}(y)$	370.26	${\hat{F}}_{9}^{\left(10\right)}(y)$	428.78

**Table 14:** PREs of distribution function estimators using Population II.

Estimator	Value	Estimator	Value	Estimator	Value	Estimator	Value	Estimator	Value
${\hat{F}}_{1}(y)$	100	${\hat{F}}_{6}^{\left(1\right)}(y)$	136.01	${\hat{F}}_{7}^{\left(1\right)}(y)$	454.79	${\hat{F}}_{8}^{\left(1\right)}(y)$	464.37	${\hat{F}}_{9}^{\left(1\right)}(y)$	502.91
${\hat{F}}_{2}(y)$	427.32	${\hat{F}}_{6}^{\left(2\right)}(y)$	132.94	${\hat{F}}_{7}^{\left(2\right)}(y)$	454.78	${\hat{F}}_{8}^{\left(2\right)}(y)$	464.36	${\hat{F}}_{9}^{\left(2\right)}(y)$	502.90
${\hat{F}}_{3}(y)$	26.53	${\hat{F}}_{6}^{\left(3\right)}(y)$	139.80	${\hat{F}}_{7}^{\left(3\right)}(y)$	454.80	${\hat{F}}_{8}^{\left(3\right)}(y)$	464.38	${\hat{F}}_{9}^{\left(3\right)}(y)$	502.92
${\hat{F}}_{4}(y)$	454.24	${\hat{F}}_{6}^{\left(4\right)}(y)$	133.38	${\hat{F}}_{7}^{\left(4\right)}(y)$	454.78	${\hat{F}}_{8}^{\left(4\right)}(y)$	464.36	${\hat{F}}_{9}^{\left(4\right)}(y)$	502.90
${\hat{F}}_{5}(y)$	454.73	${\hat{F}}_{6}^{\left(5\right)}(y)$	140.06	${\hat{F}}_{7}^{\left(5\right)}(y)$	454.80	${\hat{F}}_{8}^{\left(5\right)}(y)$	464.38	${\hat{F}}_{9}^{\left(5\right)}(y)$	502.90
		${\hat{F}}_{6}^{\left(6\right)}(y)$	140.57	${\hat{F}}_{7}^{\left(6\right)}(y)$	454.81	${\hat{F}}_{8}^{\left(6\right)}(y)$	464.38	${\hat{F}}_{9}^{\left(6\right)}(y)$	502.92
		${\hat{F}}_{6}^{\left(7\right)}(y)$	132.71	${\hat{F}}_{7}^{\left(7\right)}(y)$	454.78	${\hat{F}}_{8}^{\left(7\right)}(y)$	464.36	${\hat{F}}_{9}^{\left(7\right)}(y)$	502.92
		${\hat{F}}_{6}^{\left(8\right)}(y)$	144.15	${\hat{F}}_{7}^{\left(8\right)}(y)$	454.82	${\hat{F}}_{8}^{\left(8\right)}(y)$	464.39	${\hat{F}}_{9}^{\left(8\right)}(y)$	502.93
		${\hat{F}}_{6}^{\left(9\right)}(y)$	129.86	${\hat{F}}_{7}^{\left(9\right)}(y)$	454.77	${\hat{F}}_{8}^{\left(9\right)}(y)$	464.35	${\hat{F}}_{9}^{\left(9\right)}(y)$	502.89
		${\hat{F}}_{6}^{\left(10\right)}(y)$	100.10	${\hat{F}}_{7}^{\left(10\right)}(y)$	454.73	${\hat{F}}_{8}^{\left(10\right)}(y)$	464.30	${\hat{F}}_{9}^{\left(10\right)}(y)$	502.84

**Table 15:** PREs of distribution function estimators using Population III.

Estimator	Value	Estimator	Value	Estimator	Value	Estimator	Value	Estimator	Value
${\hat{F}}_{1}(y)$	100	${\hat{F}}_{6}^{\left(1\right)}(y)$	126.05	${\hat{F}}_{7}^{\left(1\right)}(y)$	211.54	${\hat{F}}_{8}^{\left(1\right)}(y)$	214.04	${\hat{F}}_{9}^{\left(1\right)}(y)$	241.14
${\hat{F}}_{2}(y)$	181.83	${\hat{F}}_{6}^{\left(2\right)}(y)$	121.46	${\hat{F}}_{7}^{\left(2\right)}(y)$	211.49	${\hat{F}}_{8}^{\left(2\right)}(y)$	213.99	${\hat{F}}_{9}^{\left(2\right)}(y)$	241.09
${\hat{F}}_{3}(y)$	29.41	${\hat{F}}_{6}^{\left(3\right)}(y)$	131.91	${\hat{F}}_{7}^{\left(3\right)}(y)$	211.61	${\hat{F}}_{8}^{\left(3\right)}(y)$	214.10	${\hat{F}}_{9}^{\left(3\right)}(y)$	241.22
${\hat{F}}_{4}(y)$	208.64	${\hat{F}}_{6}^{\left(4\right)}(y)$	122.03	${\hat{F}}_{7}^{\left(4\right)}(y)$	211.50	${\hat{F}}_{8}^{\left(4\right)}(y)$	214.00	${\hat{F}}_{9}^{\left(4\right)}(y)$	241.10
${\hat{F}}_{5}(y)$	211.39	${\hat{F}}_{6}^{\left(5\right)}(y)$	132.66	${\hat{F}}_{7}^{\left(5\right)}(y)$	211.62	${\hat{F}}_{8}^{\left(5\right)}(y)$	214.12	${\hat{F}}_{9}^{\left(5\right)}(y)$	241.23
		${\hat{F}}_{6}^{\left(6\right)}(y)$	133.40	${\hat{F}}_{7}^{\left(6\right)}(y)$	211.63	${\hat{F}}_{8}^{\left(6\right)}(y)$	214.13	${\hat{F}}_{9}^{\left(6\right)}(y)$	241.24
		${\hat{F}}_{6}^{\left(7\right)}(y)$	120.88	${\hat{F}}_{7}^{\left(7\right)}(y)$	211.49	${\hat{F}}_{8}^{\left(7\right)}(y)$	213.99	${\hat{F}}_{9}^{\left(7\right)}(y)$	241.08
		${\hat{F}}_{6}^{\left(8\right)}(y)$	139.14	${\hat{F}}_{7}^{\left(8\right)}(y)$	211.71	${\hat{F}}_{8}^{\left(8\right)}(y)$	214.21	${\hat{F}}_{9}^{\left(8\right)}(y)$	241.34
		${\hat{F}}_{6}^{\left(9\right)}(y)$	116.98	${\hat{F}}_{7}^{\left(9\right)}(y)$	211.46	${\hat{F}}_{8}^{\left(9\right)}(y)$	213.95	${\hat{F}}_{9}^{\left(9\right)}(y)$	241.05
		${\hat{F}}_{6}^{\left(10\right)}(y)$	100.08	${\hat{F}}_{7}^{\left(10\right)}(y)$	211.39	${\hat{F}}_{8}^{\left(10\right)}(y)$	213.88	${\hat{F}}_{9}^{\left(10\right)}(y)$	240.97

**Table 16:** PREs of distribution function estimators using Population IV.

Estimator	Value	Estimator	Value	Estimator	Value	Estimator	Value	Estimator	Value
${\hat{F}}_{1}(y)$	100	${\hat{F}}_{6}^{\left(1\right)}(y)$	125.53	${\hat{F}}_{7}^{\left(1\right)}(y)$	208.38	${\hat{F}}_{8}^{\left(1\right)}(y)$	210.61	${\hat{F}}_{9}^{\left(1\right)}(y)$	231.39
${\hat{F}}_{2}(y)$	179.42	${\hat{F}}_{6}^{\left(2\right)}(y)$	120.78	${\hat{F}}_{7}^{\left(2\right)}(y)$	208.34	${\hat{F}}_{8}^{\left(2\right)}(y)$	210.56	${\hat{F}}_{9}^{\left(2\right)}(y)$	231.34
${\hat{F}}_{3}(y)$	29.65	${\hat{F}}_{6}^{\left(3\right)}(y)$	131.68	${\hat{F}}_{7}^{\left(3\right)}(y)$	208.45	${\hat{F}}_{8}^{\left(3\right)}(y)$	210.68	${\hat{F}}_{9}^{\left(3\right)}(y)$	231.47
${\hat{F}}_{4}(y)$	205.48	${\hat{F}}_{6}^{\left(4\right)}(y)$	121.39	${\hat{F}}_{7}^{\left(4\right)}(y)$	208.34	${\hat{F}}_{8}^{\left(4\right)}(y)$	210.56	${\hat{F}}_{9}^{\left(4\right)}(y)$	231.35
${\hat{F}}_{5}(y)$	208.23	${\hat{F}}_{6}^{\left(5\right)}(y)$	132.28	${\hat{F}}_{7}^{\left(5\right)}(y)$	208.46	${\hat{F}}_{8}^{\left(5\right)}(y)$	210.69	${\hat{F}}_{9}^{\left(5\right)}(y)$	231.48
		${\hat{F}}_{6}^{\left(6\right)}(y)$	133.08	${\hat{F}}_{7}^{\left(6\right)}(y)$	208.47	${\hat{F}}_{8}^{\left(6\right)}(y)$	210.70	${\hat{F}}_{9}^{\left(6\right)}(y)$	231.49
		${\hat{F}}_{6}^{\left(7\right)}(y)$	120.31	${\hat{F}}_{7}^{\left(7\right)}(y)$	208.33	${\hat{F}}_{8}^{\left(7\right)}(y)$	210.55	${\hat{F}}_{9}^{\left(7\right)}(y)$	231.34
		${\hat{F}}_{6}^{\left(8\right)}(y)$	139.07	${\hat{F}}_{7}^{\left(8\right)}(y)$	208.56	${\hat{F}}_{8}^{\left(8\right)}(y)$	210.78	${\hat{F}}_{9}^{\left(8\right)}(y)$	231.59
		${\hat{F}}_{6}^{\left(9\right)}(y)$	116.30	${\hat{F}}_{7}^{\left(9\right)}(y)$	208.30	${\hat{F}}_{8}^{\left(9\right)}(y)$	210.52	${\hat{F}}_{9}^{\left(9\right)}(y)$	231.30
		${\hat{F}}_{6}^{\left(10\right)}(y)$	100.08	${\hat{F}}_{7}^{\left(10\right)}(y)$	208.23	${\hat{F}}_{8}^{\left(10\right)}(y)$	210.46	${\hat{F}}_{9}^{\left(10\right)}(y)$	231.23

We computed sample size in stratum *h*. Here we took sample sizes of 180, 180, 140 and 140 from four populations. Then we used stratified random sampling, and the MSEs (minimum) of the proposed families of estimators were computed in Eqs 56 and 62, respectively. Lastly, the adapted estimators and proposed families of estimators were compared with each other with respect to their PRE values. The PRE results are shown in Tables 13–16. In Tables 9–12, we can observe the descriptive statistics regarding the populations, strata, and sample size. From the numerical results, presented in Tables 13–16, it is observed that the PREs of all families of estimators change with the choices of a and b. It is further noted that the proposed families of estimators are more precise than the adapted distribution function estimators of Cochran [31], Murthy [32], Rao [4], Singh et al. [33] and Grover and Kaur [11], in terms of the PRE. It can be seen that, for all data sets, the second proposed family of estimators perform better than the first proposed family of estimators. We can also see a rise in the value of PREs when the value of a = 1; and b = *R*_12_; a = *R*_12_ and b = *C*_2_; a = *R*_12_ and b = *β*_2(st)_, and see a slight fall in PREs when a = 1 and b = *NF*(x)).

## 12 Conclusion

In this paper, we have proposed two new families of estimators for estimating the finite population distribution function under simple and stratified random sampling schemes. The proposed estimators required supplementary information about the sample mean and the ranks of the auxiliary variable. The biases and MSEs of the proposed families of estimators were derived using the first order approximation. Based on theoretical and numerical comparative studies, it can be concludethat the proposed families of estimators are more precise than their existing counterparts. Thus, we recommend using the sample mean and the ranks of the auxiliary variable with the proposed families of estimators for estimating the finite population distribution function under simple or stratified random sampling. It would be interesting to extend the suggested estimators to two-phase and stratified two-phase sampling schemes. Furthermore, the proposed estimators could also be generalised by utilising information about multi-auxillary variables.

## References

[1] Murthy MN. Sampling Theory and Methods. Statistical Pub 1967 Society,Calcutta.

[2] Sisodia BV, Dwivedi VK. Modified ratio estimator using coefficient of variation of auxiliary variable. Journal-Indian Society of Agricultural Statistics. 1981.

[3] Srivastava SK, Jhajj HS. A class of estimators of the population mean using multiauxiliary information. Calcutta Statistical Association Bulletin. 1983 3;32(1–2):47–56.

[4] Rao TJ. On certail methods of improving ration and regression estimators. Communications in Statistics-Theory and Methods. 1991 1 1;20(10):3325–40.

[5] Upadhyaya LN, Singh HP. Use of transformed auxiliary variable in estimating the finite population mean. Biometrical Journal: Journal of Mathematical Methods in Biosciences. 1999 9;41(5):627–36.

[6] Singh S. Advanced Sampling Theory With Applications: How Michael"" Selected"" Amy. Springer Science & Business Media; 2003.

[7] Kadilar C, Cingi H. Estimator of a population mean using two auxiliary variables in simple random sampling. International Mathematical Journal. 2004;5:357–67.

[8] Kadilar C, Cingi H. Improvement in estimating the population mean in simple random sampling. Applied Mathematics Letters. 2006 1 1;19(1):75–9.

[9] Gupta S, Shabbir J. On improvement in estimating the population mean in simple random sampling. Journal of Applied Statistics. 2008 5 1;35(5):559–66.

[10] Grover LK, Kaur P. Ratio type exponential estimators of population mean under linear transformation of auxiliary variable: theory and methods. South African Statistical Journal. 2011 1 1;45(2):205–30.

[11] Grover LK, Kaur P. A generalized class of ratio type exponential estimators of population mean under linear transformation of auxiliary variable. Communications in Statistics-Simulation and Computation. 2014 1 1;43(7):1552–74.

[12] Lu J. Efficient estimator of a finite population mean using two auxiliary variables and numerical application in agricultural, biomedical, and power engineering. Mathematical Problems in Engineering. 2017 1 1;2017. 10.1155/2017/2696108 29578548 PMC5865224

[13] Muneer S, Shabbir J, Khalil A. Estimation of finite population mean in simple random sampling and stratified random sampling using two auxiliary variables. Communications in Statistics-Theory and Methods. 2017 3 4;46(5):2181–92.

[14] Shabbir J, Gupta S. Estimation of finite population mean in simple and stratified random sampling using two auxiliary variables. Communications in Statistics-Theory and Methods. 2017 10 18;46(20):10135–48.

[15] Gupta RK, Yadav SK. Improved estimation of population mean using information on size of the sample. American Journal of Mathematics and Statistics. 2018;8(2):27–35.

[16] Haq A, Khan M, Hussain Z. A new estimator of finite population mean based on the dual use of the auxiliary information. Communications in Statistics-Theory and Methods. 2017 5 3;46(9):4425–36.

[17] Chambers RL, Dunstan R. Estimating distribution functions from survey data. Biometrika. 1986 12 1;73(3):597–604.

[18] Rao JN, Kovar JG, Mantel HJ. On estimating distribution functions and quantiles from survey data using auxiliary information. Biometrika. 1990 6 1:365–75.

[19] Rao JN. Estimating totals and distribution functions using auxiliary information at the estimation stage. Journal of Official Statistics. 1994 6 1;10(2):153.

[20] Kuk AY. A kernel method for estimating finite population distribution functions using auxiliary information. Biometrika. 1993 6 1;80(2):385–92.

[21] Ahmed MS. and Abu-Dayyeh W. Estimation of finite-population distribution function using multivariate auxiliary information. Statistics in Transition. 2001 5(3):501–507.

[22] Rueda M, Martínez S, Martínez H, Arcos A. Estimation of the distribution function with calibration methods. Journal of statistical planning and inference. 2007 2 1;137(2):435–48.

[23] Singh HP, Singh S, Kozak M. A family of estimators of finite-population distribution function using auxiliary information. Acta applicandae mathematicae. 2008 11 1;104(2):115–30.

[24] Yaqub M, Shabbir J. Estimation of population distribution function in the presence of non-response. Hacettepe Journal of Mathematics and Statistics. 2018 4 1;47(2):471–511.

[25] Chen F, Chen S. Injury severities of truck drivers in single-and multi-vehicle accidents on rural highways. Accident Analysis & Prevention. 2011 9 1;43(5):1677–88. 21658494 10.1016/j.aap.2011.03.026

[26] Zeng Q, Wen H, Huang H, Pei X, Wong SC. A multivariate random-parameters Tobit model for analyzing highway crash rates by injury severity. Accident Analysis & Prevention. 2017 2 1;99:184–91. 27914307 10.1016/j.aap.2016.11.018

[27] Dong B, Ma X, Chen F, Chen S. Investigating the differences of single-vehicle and multivehicle accident probability using mixed logit model. Journal of Advanced Transportation. 2018 1 1;2018. 10.1155/2018/7905140 32908973 PMC7477825

[28] Chen F, Chen S, Ma X. Analysis of hourly crash likelihood using unbalanced panel data mixed logit model and real-time driving environmental big data. Journal of safety research. 2018 6 1;65:153–9. 10.1016/j.jsr.2018.02.010 29776524

[29] Zeng Q, Guo Q, Wong SC, Wen H, Huang H, Pei X. Jointly modeling area-level crash rates by severity: a Bayesian multivariate random-parameters spatio-temporal Tobit regression. Transportmetrica A: Transport Science. 2019 11 29;15(2):1867–84.

[30] Zeng Q, Wen H, Wong SC, Huang H, Guo Q, Pei X. Spatial joint analysis for zonal daytime and nighttime crash frequencies using a Bayesian bivariate conditional autoregressive model. Journal of Transportation Safety & Security. 2020 4 20;12(4):566–85. 10.4271/2016-01-1439 27648455 PMC5026383

[31] Cochran WG. The estimation of the yields of cereal experiments by sampling for the ratio of grain to total produce. The journal of agricultural science. 1940 4;30(2):262–75.

[32] Murthy MN. Product method of estimation. Sankhyā: The Indian Journal of Statistics, Series A. 1964 7 1:69–74.

[33] Singh R, Chauhan P, Sawan N, Smarandache F. Improvement in estimating the population mean using exponential estimator in simple random sampling. International Journal of Statistics & Economics. 2009 3(A09):13–18

[34] Gujarati DN. Basic econometrics. Tata McGraw-Hill Education; 2009.

[35] Särndal CE. Methods for estimating the precision of survey estimates when imputation has been used. Survey methodology. 1992;18(2):241–52.

[36] Koyuncu N, Kadilar C. Ratio and product estimators in stratified random sampling. Journal of statistical planning and inference. 2009 8 1;139(8):2552–8.

[37] Kadilar C, Cingi HB. Ratio estimators in stratified random sampling. Biometrical Journal: Journal of Mathematical Methods in Biosciences. 2003 3;45(2):218–25.

